# Impact of charge switching stimuli on supramolecular perylene monoimide assemblies†
†Electronic supplementary information (ESI) available. See DOI: 10.1039/c8sc05595e


**DOI:** 10.1039/c8sc05595e

**Published:** 2019-05-14

**Authors:** Adam Dannenhoffer, Hiroaki Sai, Dongxu Huang, Benjamin Nagasing, Boris Harutyunyan, Daniel J. Fairfield, Taner Aytun, Stacey M. Chin, Michael J. Bedzyk, Monica Olvera de la Cruz, Samuel I. Stupp

**Affiliations:** a Department of Materials Science and Engineering , 2220 Campus Drive , Evanston , IL 60208 , USA; b Department of Chemistry , Northwestern University , 2145 Sheridan Road , Evanston , IL 60208 , USA . Email: s-stupp@northwestern.edu; c Department of Medicine , Northwestern University , 676 N St. Clair , Chicago , Illinois 60611 , USA; d Simpson Querrey Institute , Northwestern University , 303 E. Superior , Chicago , Illinois 60611 , USA; e Department of Biomedical Engineering , Northwestern University , 2145 Sheridan Road , Evanston , IL 60208 , USA; f Department of Physics and Astronomy , Northwestern University , 2145 Sheridan Road , Evanston , IL 60208 , USA

## Abstract

The development of stimuli-responsive amphiphilic supramolecular nanostructures is an attractive target for systems based on light-absorbing chromophores that can function as photosensitizers in water. We report here on a water soluble supramolecular carboxylated perylene monoimide system in which charge can be switched significantly by a change in pH. This was accomplished by substituting the perylene core with an ionizable hydroxyl group. In acidic environments, crystalline supramolecular nanoribbons with dimensions on the order of 500 × 50 × 2 nm form readily, while in basic solution the additional electrostatic repulsion of the ionized hydroxyl reduces assemblies to very small dimensions on the order of only several nanometers. The HOMO/LUMO levels were also found to be sensitive to pH; in acidic media the HOMO/LUMO levels are –5.65 and –3.70 eV respectively *versus* vacuum, whereas is in basic conditions they are –4.90 and –3.33 eV, respectively. Utilizing the assemblies as photosensitizers in photocatalytic production of hydrogen with [Mo_3_S_13_]^2–^ as a catalyst at a pH of 4, H_2_ was generated with a turnover number of 125 after 18 hours. Charge switching the assemblies at a pH of 9–10 and using an iron porphyrin catalyst, protons could again be reduced to hydrogen and CO_2_ was reduced to CO with a turnover number of 30. The system investigated offers an example of dynamic photosensitizing assemblies that can drive reactions in both acidic and basic media.

## Abstract 2

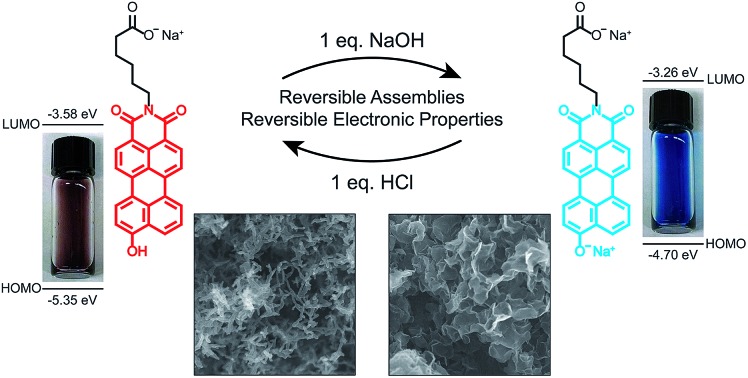
A switchable perylene monoimide which undergoes reversible morphological and electronic changes controlled by the ionization state of the phenolic oxygen.

## Introduction

Supramolecular assembly involving weak interactions yields nanostructures whose properties and functions are highly susceptible to stimuli.^1^–^3^ Many supramolecular materials have been developed in which their nanostructure morphologies can be controlled in response to solvent polarity,^4^,^5^ ionic strength,^6^ or reversibly using pH.^7^ Organic chromophore assemblies are often investigated for functions that take advantage of their light absorbing^8^ or electrically conductive properties.^9^ If an assembly of chromophores is designed to be pH-responsive, it is possible to control both nanoscale morphology and electronic properties with a single switch. This ability to tune the properties of supramolecular nanostructures composed from optically and electronically active building blocks is important for them to function as components in organic electronic devices^10^ or within photocatalytic systems.^6^,^11^

Rylene dyes, such as perylene diimide (PDI) and perylene monoimide (PMI), are excellent candidates for the design of stimuli-responsive chromophore materials due to their tunable optical and redox properties, high chemical stability, and their strong tendency to assemble into supramolecular structures.^12^–^15^ The optical and redox properties of rylene dyes can be modified by substitution of electron donating and withdrawing groups on the aromatic core.^13^,^16^–^23^ A wide range of different chromophores can be obtained through direct covalent modification, although these changes permanently fix absorbance and redox properties of the dyes. However, if a substituent group is pH-sensitive, the chromophore can possess distinctly different electronic properties depending on the solution conditions.^24^–^26^ Therefore, it is interesting to investigate how chromophore design can modulate not only the electronic structure but also the self-assembly behavior of an amphiphile used in organic devices.

The aggregation of rylene dyes is known to be highly dependent on the nature of their substituents. Various nanoscale morphologies such as fibers, ribbons, and micelles have been observed in aggregates formed using PDI and PMI molecules by controlling the substituents at the imide position,^11^,^27^–^29^ as well as directly attached to the aromatic core.^30^,^31^ Self-assembly in water requires amphiphilic compounds, and examples are known in which this occurs by formation of charged groups as a result of pH changes.^32^–^34^ In molecules containing multiple ionizable groups, reversible changes of nanoscale morphology have been reported by altering pH.^7^,^35^–^38^ Previous work from our laboratory has demonstrated that PMIs with a single pendant carboxylic acid can trigger the formation of supramolecular assemblies in water.^39^,^40^ While deprotonation of the carboxylic acid does promote solubility and self-assembly, it does not allow reversible control of nanostructure morphology. The addition of a pH-sensitive functional group on the aromatic core should allow the possibility of multiple ionization states, and more diverse nanostructures in response to changes in pH.^41^,^42^

We report here on an amphiphilic perylene monoimide (PMI) containing an ionizable hydroxyl group attached to the 9-position of PMI. We investigated the supramolecular chemistry and electronic properties of the resulting nanostructures when the 9-position hydroxyl group is either ionized or protonated. A combination of absorbance spectroscopy, ultraviolet photoelectron spectroscopy, and density functional theory were used to characterize the electronic structure of our pH-sensitive assemblies. Nanostructure morphology was characterized using X-ray scattering, microscopy, and molecular dynamics simulations.

## Results and discussion

### Absorbance behavior

**Fig. 1:**
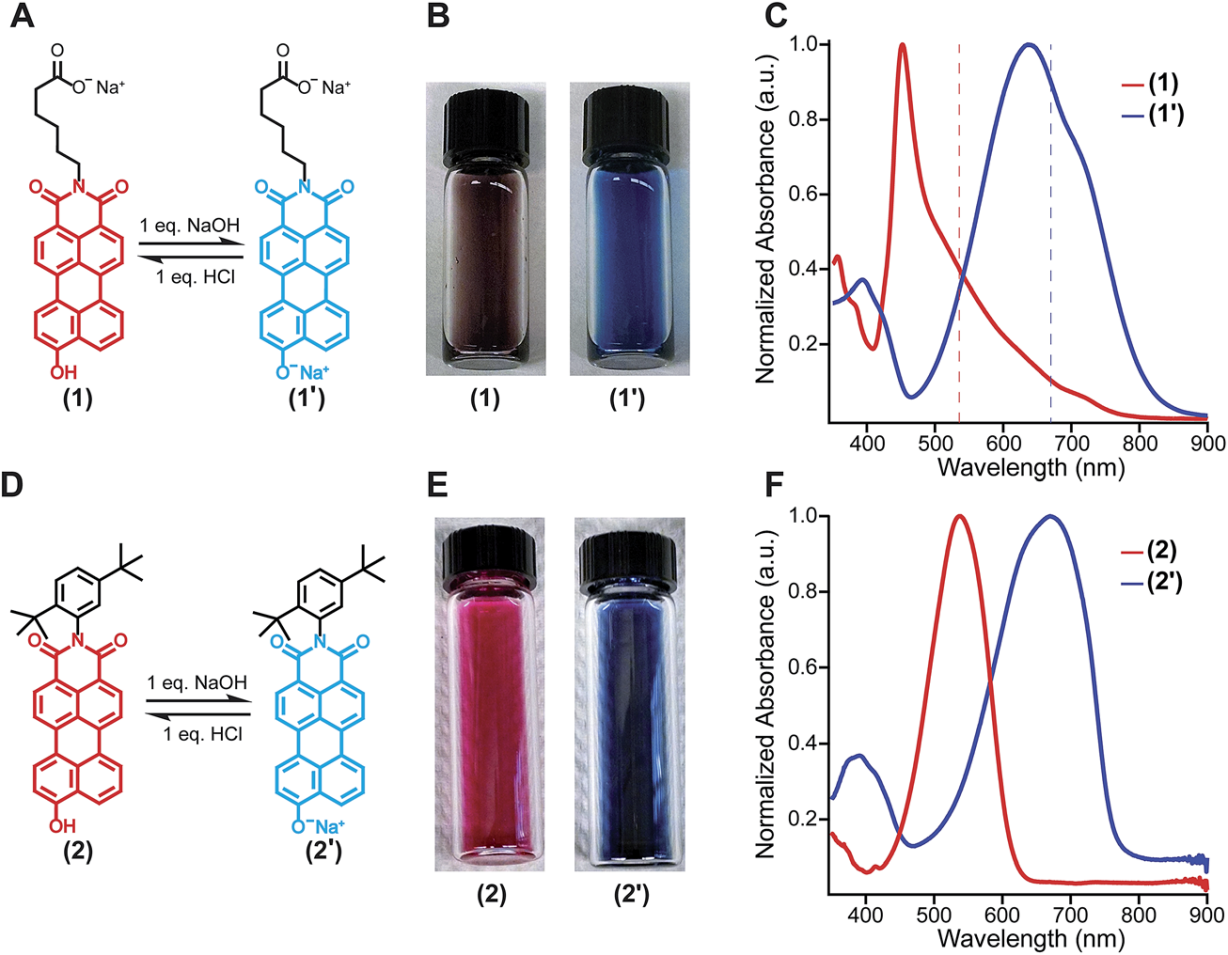
(A) Chemical structures showing the reversible ionization of **1**. (B) Photographs of aqueous solutions of protonated (**1**, left) and deprotonated (**1′**, right) (C) UV-vis absorbance spectra of **1** (8.7 mM) in water. (D) Chemical structures showing the reversible change in ionization state of **2**. (E) Photographs of aqueous solutions of **2** in 9 : 1 DCM/MeOH while protonated (**2**, left) and deprotonated (**2′**, right) (F) UV-vis absorbance spectra of **2** in 9 : 1 DCM/MeOH (0.87 mM).

The amphiphilic hydroxylated perylene monoimide **1** was synthesized using a palladium cross-coupling of water to 9-bromo-*N*-(methyl hexanoate) perylene-3,4-dicarboximide, followed by hydrolysis of the methyl ester to impart water solubility (see Scheme 1 in ESI†). Dissolution of **1** in water with two equivalents of sodium hydroxide led to the formation of a deep blue solution (Fig. 1B, right). Upon addition of one equivalent of hydrochloric acid, the solution rapidly turned reddish brown (Fig. 1B, left). These color changes in response to acid or base are indicated by the 187 nm shift in the maximum absorbance (*λ*_max_) under the two conditions tested (Fig. 1C). After five cycles of acid/base addition, the absorbance spectra were re-recorded and showed identical behavior to the first cycle demonstrating the reversibility of this system (ESI Fig. 4†). When dissolved in water, **1** can exist in two distinct protonation states, depending on the solution pH (Fig. 1A). When protonated, the absorbance spectrum of **1** exhibits a sharp and highly blue-shifted peak (*λ*_max_ 460 nm) similar to that of previously reported amphiphilic PMIs that form crystalline nanostructures in aqueous solution.^6^,^39^,^40^ In contrast, the absorbance spectrum when the hydroxyl group is deprotonated (**1′**) displays a broad peak (*λ*_max_ 643 nm) which resembles previously reported non-crystalline PMI nanostructures.^6^,^43^ The p*K*_a_ of the hydroxyl group was determined to be ∼9 by tracking the relative absorbance at 460 and 643 nm after incubating **1** in various buffers with pH values ranging from 3 to 11 (ESI Fig. 5†). Between pH 8 and 10 a sharp decrease in the absorbance at 460 nm is coupled to an increase in the absorbance at 643 nm indicating the transition from the protonated to the deprotonated compound.

To confirm the hydroxyl group ionization state is responsible for the observed color change in response to pH, we collected spectroscopic data on the analogous compound with a bulky imide substituent (**2**), which remains monomeric in organic solvents such as DCM, DMSO, or DMF (Fig. 1D). Compound **2** was found to exhibit a similar blue to red color change as its water-soluble analog, again demonstrated by a 133 nm shift in the *λ*_max_ in response to the addition of base (Fig. 1E and F). Interestingly, while **2** did not show any response to changes in solvent polarity, its conjugate base (**2′**) exhibited significant solvatochromism (ESI Fig. 6†). This observation is consistent with the behavior of other organic dyes bearing hydroxyl substituents.^44^,^45^ While we have demonstrated the protonation state of the hydroxyl group strongly affects the absorbance of **1**, it is also important to note supramolecular assembly also influences the absorbance spectrum. In water, **1** displays a sharp peak that is 85 nm blue-shifted compared to its monomer. The absorbance of **1′** displays a small blue shift in *λ*_max_, but a similar broad featureless peak shape. The differences in absorbance line shape indicate the assembly state of compound **1** and **1′** are different in aqueous solution.

### Nanostructure morphology

**Fig. 2:**
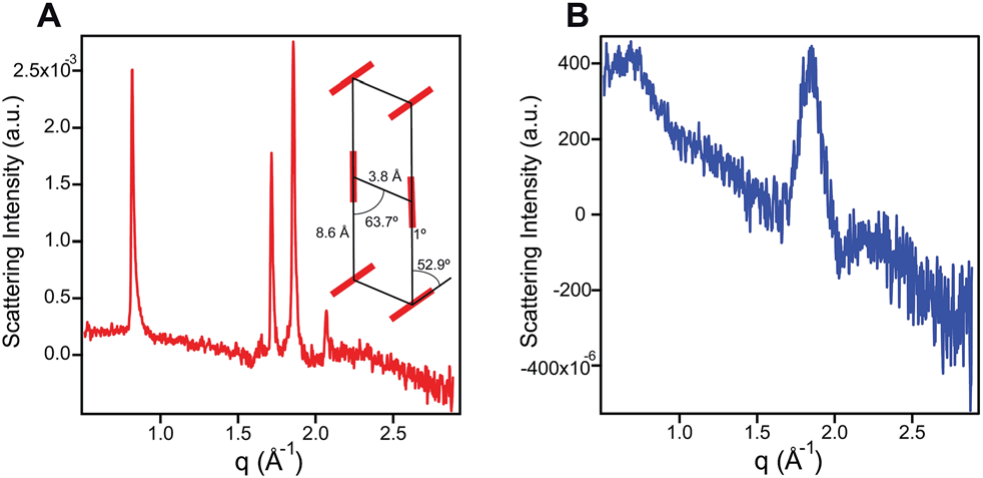
(A) Wide angle X-ray scattering scan for **1** (7.25 mM) in water. The inset shows a herringbone lattice which represents the best numerical fit to the scattering data. Crystal domain size was estimated to be 476 Å corresponding to 142 stacked units using the Debye–Scherrer equation. (B) Wide angle X-ray scattering scan for **1′** (7.25 mM) in water showing a broad peak corresponding to a 3.42 Å *d*-spacing, and a domain size of 33 Å or 11 stacked units.

In order to probe the differences in assembly state between **1** and **1′**, solution phase wide-angle X-ray scattering (WAXS) experiments were performed on 7.25 mM solutions of each compound. The scattering trace for **1** revealed the presence of sharp and intense peaks suggesting a high degree of order within the nanostructures present in solution (Fig. 2A). Numerical modeling, developed in earlier work,^39^ of the scattering peaks show that **1** assembles into a 2D parallelepiped unit cell with molecules arranged in a herringbone lattice and a domain size of 476 Å (Fig. 2A, inset (see ESI† for details on modeling)). In contrast, the WAXS trace for ionized **1′** showed only a single broad and less intense scattering maximum, thus revealing a more disordered packing of molecules within the solution (Fig. 2B). This scattering peak corresponds to a real space distance of roughly 3.42 Å, which we attribute to π–π stacking among **1′** molecules, and a domain size of 33 Å estimated using the Debye–Scherrer equation. The above scattering data suggests that when protonated, **1** can form larger nanostructures with a high degree of internal order, while after deprotonation **1′** forms only small, more disordered, structures.

**Fig. 3:**
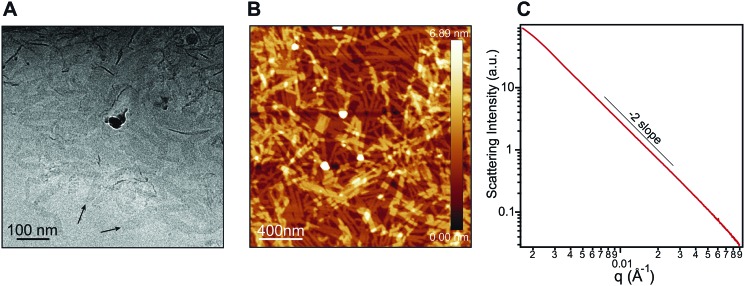
(A) Cryo-TEM of an aqueous solution of **1** (7.25 mM). (B) AFM of **1** (7.25 mM) spin coated from water onto freshly cleaved mica. (C) Small angle X-ray scattering (SAXS) patterns of **1** (7.25 mM) in water showing a Porod slope of –2, indicating the presence of 2D nanostructures over the length scale probed here.

Nanostructure morphology for each protonation state was characterized using a variety of microscopy and X-ray techniques. Distinct self-assembled morphologies were observed for each protonation state as expected from the differences in molecular packing and optical absorbance. Cryogenic transmission electron microscopy (cryo-TEM) of **1** revealed the presence of supramolecular nanoscale ribbons consistent with other previously reported amphiphilic PMI molecules (see Fig. 3A). Atomic force microscopy (AFM) was also used to confirm the ribbon morphology and determine the approximate dimensions of the nanostructures. The supramolecular ribbons were found to be roughly 50 nm wide, 300–700 nm long, and ∼2 nm in thickness, consistent with an antiparallel packing of the PMI aromatic groups within the nanoribbon (Fig. 3B). The small angle X-ray scattering (SAXS) profile of **1** in aqueous solution showed a –2 slope in the Porod region, supporting the ribbon morphology observed by microscopy^46^,^47^ (Fig. 3C). After deprotonation, **1′** did not show any significant nanostructure formation by cryo-TEM, AFM, or SAXS consistent with the absorbance and WAXS data, which suggests only small aggregates can form under these conditions (ESI Fig. 8, 10 and 19†).

**Fig. 4:**
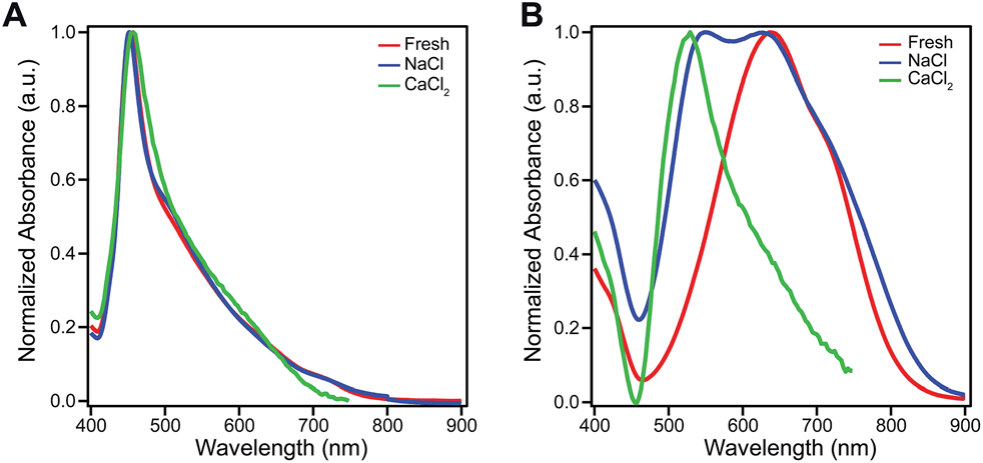
(A) Normalized UV-vis absorbance spectra of **1** (7.25 mM), freshly dissolved in aqueous solution (red curve), in the same solution but containing 50 mM NaCl and annealed at 80 °C for 30 minutes (blue curve), and after gelation by adding 43 mM CaCl_2_ (green curve). (B) UV-vis absorbance spectra of compound **1′** under the same conditions described in (A).

We hypothesize the dramatic differences in absorbance spectral shape, molecular packing, and morphology are due to increased electrostatic repulsion present in **1′**. To promote supramolecular assembly, solutions of **1** and **1′** were annealed in the presence of various salts to screen electrostatic repulsion among molecules and allow the systems to overcome any kinetic trap states. Fig. 4A shows that the optical properties of **1** are insensitive to either annealing or salt. In contrast, the optical absorbance of **1′** was found to be highly sensitive to both salt and annealing. For example, when exposed to CaCl_2_ the absorbance of **1′** becomes highly blue-shifted and displays a similar spectral shape to that of **1** (Fig. 4B). It is also important to note that both **1** and **1′** form weak hydrogels when exposed to CaCl_2_ due to the nanostructures becoming entangled after repulsion among their negatively charged surfaces is reduced through electrostatic screening. SEM imaging revealed that **1** maintained the morphology observed without added salt, while **1′** developed a sheet-like morphology only after Ca^2+^ exposure (ESI Fig. 14 and 15†). Grazing incidence wide-angle X-ray scattering (GIWAXS) of dropcast samples of **1′** incubated with CaCl_2_ revealed similar scattering peaks for both the protonated and deprotonated molecules (ESI Fig. 13†). Evidently, the presence of the ionized hydroxyl does have a strong influence on the self-assembly of this PMI amphiphile, and the addition of screening ions can be used to induce crystallization.

### Molecular dynamic simulations

**Fig. 5:**
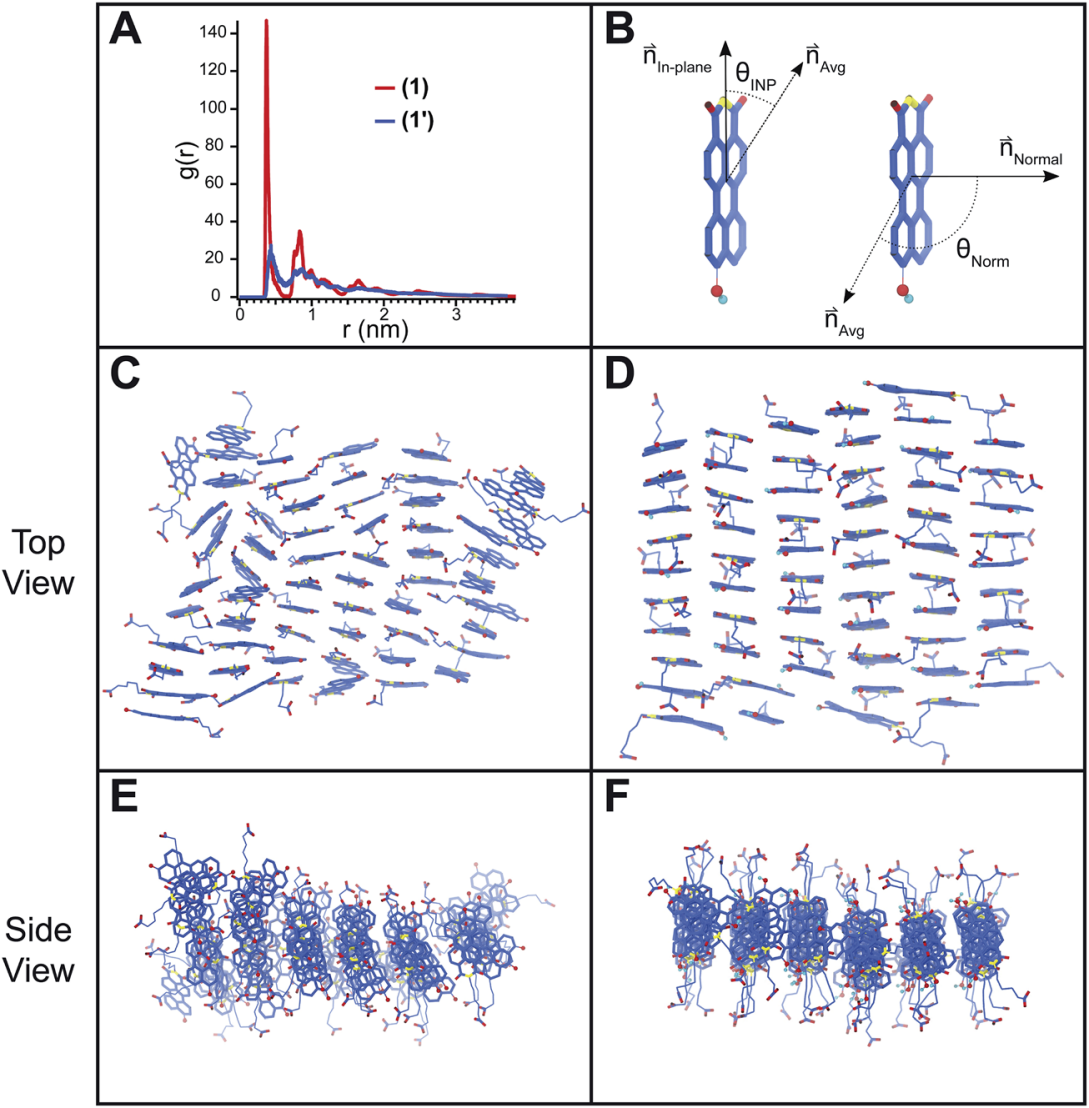
Snapshots of MD simulations on pre-assembled PMI nanoscale ribbons. (A) Radial distribution function of the PMI center to center distance for both **1** and **1′**. (B) Schematic of the two vectors (*n*_in-plane_, *n*_normal_) used to describe the rotational order of PMI assemblies. (C) Top view of **1′** after equilibrium is reached. (D) Top view of **1** after equilibrium is reached. (E) Side view of **1′**. (F) Side view of **1**. Explicit water molecules used in the simulations are omitted from snapshots for clarity.

Atomistic molecular dynamics (MD) simulations were conducted on pre-assembled ribbons of **1** and **1′** to gain further insight into the packing behavior of the two species. These simulations were particularly useful in understanding the packing of **1′** for which spectroscopic and scattering data do not provide insight into the packing at a molecular level. Sixty PMI molecules were arranged into ribbons with a 0.36 × 0.9 nm unit cell and then allowed to relax inside of an 8 × 8 × 8 nm simulation box with explicit water and ions. Snapshots of the simulation results are shown in Fig. 5 for both **1** and **1′**. The ribbon structure and internal order of the **1′** are significantly compromised when the simulation reaches equilibrium. PMI molecules can be seen to both translate out of the ribbon's plane and rotate with respect to their initial position (Fig. 5C and E). In contrast, **1** maintains its ribbon morphology and its high degree of internal order (Fig. 5D and F). A plot of the radial distribution function (RDF) for PMI center to center distance reveals a sharp peak corresponding to a 3.72 Å spacing in the case of the **1**, which is similar to the π–π stacking distance observed in X-ray diffraction experiments (Fig. 5A). Conversely, the RDF of **1′** shows a weak/broad peak indicating the lower degree of transitional order caused by the breakdown in ribbon structure.

The rotational order for each simulation was also quantified by considering two vectors (normal, in-plane) associated with each PMI molecule (Fig. 5B). The angle between these vectors and their respective average (*θ*_Norm_ or *θ*_INP_) at the end of each simulation was determined and used to calculate an orientation parameter (OP = <(3(cos^2^(*θ*_Norm/INP_))–1))/2> where an OP of 1 represents perfect alignment, and a value of 0 indicates a random arrangement (see ESI† MD Methods). The OPs for a simulation of **1** were found to be 0.96 and 0.87 for the normal and in-plane vectors, respectively. Both values decreased in the case of the deprotonated **1′** to 0.78 and 0.68 for the normal and in-plane vectors, indicating a significant reduction in rotational order when the hydroxyl group is deprotonated. Simulations were also run in the presence of excess salt ions (Na^+^, Ca^2+^) to determine if an increase in ionic strength would lead to assemblies with superior order (ESI Fig. 20†). Addition of both NaCl and CaCl_2_ caused an increase in the order of **1′** assemblies (CaCl_2_: OP_Norm_ = 0.88, OP_INP_ = 0.79; NaCl: OP_Norm_ = 0.88, OP_INP_ = 0.74), while the presence of NaCl had little effect on **1** (NaCl: OP_Norm_ = 0.96, OP_INP_ = 0.85). These simulations support our absorption (Fig. 3B) and GIWAXS (ESI Fig. 13†) data indicating that **1′** is only able to form ordered nanostructures under highly charge-screening conditions and provides molecular insight into the translational and rotational motion of the **1′** molecules.

### Electronic structure and photocatalysis

**Fig. 6:**
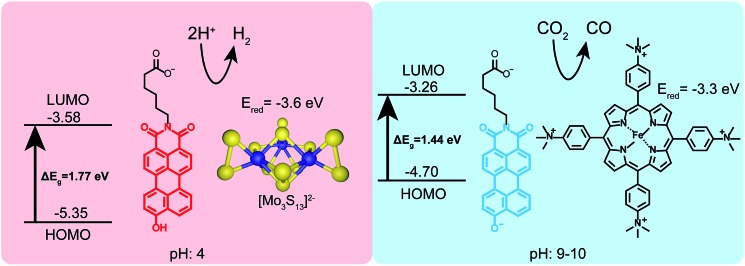
Schematic showing the HOMO/LUMO levels and two different photocatalytic reactions driven by **1** under different pH conditions. Under acidic conditions (pH 4 ascorbic acid buffer) the hydroxyl group remains protonated and **1** can photosensitize a proton reduction catalyst ([Mo_3_S_13_]^2–^). Under basic conditions (pH 9–10 ascorbate) **1′** can sensitize an iron-porphyrin catalyst which selectively reduces CO_2_ to CO.

Given that PMI assemblies can act as photosensitizers, we sought to examine how the ionization state of **1** affects its electronic energy levels. Ultraviolet photoelectron spectroscopy (UPS) was used to determine the HOMO energy level for both the protonated and deprotonated assemblies (ESI Fig. 18†). The bandgap for each protonation state was determined from absorbance data to be 1.72 eV and 1.44 eV for **1** and **1′**, respectively. The energy level diagram in Fig. 6 shows the relative location of both the HOMO and LUMO of each state *versus* vacuum, where the LUMO has been calculated by adding the band gap energy to the HOMO energy level. After deprotonation, the HOMO energy was observed to increase by 0.65 eV while the LUMO energy only increases by 0.32 eV. These results indicate we can alter significantly both the HOMO and LUMO energy levels of the PMI supramolecular nanostructures with simple acid/base chemistry, an effect not commonly possible when using chromophore assemblies. We conclude that the protonation state of the hydroxylated PMI controls both supramolecular morphology and the electronic energy levels in these assemblies.

The electron donating ability of the hydroxyl substituent is substantially increased after deprotonation based on the *σ*_m_ and *σ*_p_ Hammett parameters, and we hypothesize this is the cause of the changes in energy level.^48^ To confirm this hypothesis and decouple any effects of supramolecular self-assembly, the energy levels for non-assembled **2** and **2′** were also determined using a combination of UPS and absorbance spectroscopy (ESI Fig. 21†). Compound **2** exhibits a similar increase in both the HOMO and LUMO energies upon deprotonation. Since these PMIs do not aggregate into distinct nanostructures, we can attribute the changes in energy levels primarily to the differences in electron donating ability of the protonated and deprotonated hydroxyl group. Comparing the energy levels of compound **1** (assembled into supramolecular nanostructures) and **2** (non-assembled) we did observe an increase of about 0.25 eV in the HOMO energy of the PMIs when assembled. Since this change is small compared to the differences in energy between the two protonation states, we conclude self-assembly is not the dominant factor in controlling energy levels of these PMI assemblies, but instead only contributes to small changes in the HOMO and LUMO in this system.

Density functional theory (DFT) calculations were performed to support the difference in HOMO/LUMO energies observed experimentally upon deprotonation, and to determine the structure and energy of the hydroxylated PMI's frontier orbitals using the 6-311++(d,p) basis set. The results for a protonated PMI are in excellent agreement between the experimental and calculated value of the HOMO energy (Table 1). However, our calculations overestimated the LUMO and bandgap energies, a result which has been previously reported for calculations performed on other rylene dyes.^49^ Calculations carried out on a deprotonated PMI also estimate the experimental HOMO value well, but again overestimate the bandgap and LUMO level. Examining the frontier orbitals reveals that in both the protonated and deprotonated state the phenolic oxygen is involved in the orbital structure (ESI Fig. 22†). After deprotonation, the electronic density in the lobes around the phenolic oxygen was increased due to the increase electronic donating ability of the oxygen anion.

**Table 1:** HOMO/LUMO energies, band gap, and molecular dipole moment of hydroxyl-PMI monomer determined from DFT calculations and experimental values for **2**

Species	*μ* [debye]	*E*_HOMO_ [eV]		*E*_LUMO_ [eV]		Bandgap [eV]	
		DTF	EXP	DFT	EXP	DFT	EXP
PMI-OH	7.70	–5.52	–5.65	–2.98	–3.70	2.54	1.95
PMI-O^–^Na^+^	17.72	–4.78	–4.90	–2.59	–3.33	2.19	1.57

Finally, to demonstrate the utility of this pH-responsive PMI system as a photosensitizer, both protonation states were tested in photocatalytic proton and carbon dioxide reduction experiments (Fig. 6). In a typical proton reduction experiment, molybdenum sulfur clusters (NH_4_)_2_[Mo_3_S_13_] (3.5 μM) were added to PMI samples in pH 4 ascorbic acid buffers and irradiated for 18 hours (see ESI† for details). Under these conditions, nanostructures of **1** are present and act as the photosensitizer in our reaction scheme, reducing the catalyst after first having its exciton reductively quenched by ascorbic acid. Although the redox potential of the radical anion of **1** could not be directly measured with cyclic voltammetry the nanostructures are able to photosensitize the [Mo_3_S_13_]^2–^ cluster, as evidenced by the production of H_2_ gas with an average turnover number (TON) of 125 ± 31. This represents a rather modest efficiency for a PMI based system due to the inability of compound **1** to support charge transfer exciton formation, a critical component for efficient photocatalysis.^39^,^40^,^43^,^50^ Photosensitization was also tested using a recently developed water-soluble iron porphyrin (Fe-*p*-TMA) catalyst.^51^ Fe-*p*-TMA has been shown to electrochemically reduce CO_2_ to CO at potentials as low as –1.1 volts (SCE) in solutions with pH 7 and reduce protons to H_2_ in acidic media. Experiments have also shown the ability to photosensitize this Fe-*p*-TMA with organic sensitizers.^52^ Substituting Fe-*p*-TMA (6 μM) for (NH_4_)_2_[Mo_3_S_13_] in the experimental conditions above yielded no H_2_ production. This indicates the radical anion of **1** is unable to sensitize Fe-*p*-TMA. However, under basic conditions (pH 9–10) using **1′**, which possesses a higher redox potential, Fe-*p*-TMA can be sensitized. In one experiment **1′** (0.87 mM) was dissolved in acetonitrile (800 μL) with triethylamine (100 μL), Fe-*p*-TMA (6 μM) and irradiated for 48 hours under Ar. H_2_ was produced with a TON of 23 ± 6. If **1′** (0.87 mM) is dissolved in sodium ascorbate (0.5 M) with Fe-*p*-TMA (6 μM) and irradiated under CO_2_ for 48 hours CO could be observed with an estimated average TON of 30 ± 8 (see ESI† for details). This experiment shows that same chromophore can photosensitize different catalysts under different pH conditions which also demand different redox potentials.

## Conclusions

We have designed and synthesized a novel water-soluble perylene monoimide amphiphile with a hydroxyl group, which exhibits reversible structural, optical, and electrochemical properties in response to pH. The increase in electron donating ability of the deprotonated hydroxyl anion compared to the protonated analog results in a substantial increase in the energy levels of the dye and a significant reduction in the bandgap. The addition of a second negative charge to the PMI core after deprotonation hinders the formation of extended supramolecular structures, while crystalline nanoribbons can be observed when the hydroxyl group is protonated. This chromophore was also shown to participate in the photocatalytic reduction of protons or carbon dioxide under acidic and basic conditions, respectively, demonstrating the ability to control the photosensitizing properties of supramolecular assemblies.

## Conflicts of interest

There are no conflicts to declare.

## Supplementary Material

Supplementary information
