# Supplementary material for: Impact of charge switching stimuli on supramolecular perylene monoimide assemblies
Source: Chem Sci. 2019 May 14;10(22):5779–86. doi: 10.1039/c8sc05595e (PMC6568310; doi:10.1039/c8sc05595e)
Supplement: Supplementary file 1 [file SC-010-C8SC05595E-s001.pdf]

Electronic Supplementary Material (ESI) for Chemical Science.
This journal is © The Royal Society of Chemistry 2019

Impact of Charge Switching Stimuli on Supramolecular Perylene Monoimide Assemblies

Supporting Information

Adam Dannenhoffer[1], Hiroaki Sai[1,4], Dongxu Huang[1], Benjamin Nagasing[2], Boris Harutyunyan, Daniel J Fairfield[1], Taner Aytun[1], Stacey M Chin[2] Michael J. Bedzyk[1,6], Monica Olvera de la Cruz[1,2], Samuel I. Stupp[1,2,3,4,5*]

[1]Department of Materials Science and Engineering, 2220 Campus Drive, Evanston, IL 60208, USA.

[2]Department of Chemistry, Northwestern University, 2145 Sheridan Road, Evanston, IL 60208, USA.

[3]Department of Medicine, Northwestern University, 676 N. St. Clair, Chicago, Illinois 60611, USA.

[4]Simpson Querrey Institute, Northwestern University, 303 E. Superior, Chicago, Illinois 60611, USA.

[5]Department of Biomedical Engineering, Northwestern University, 2145 Sheridan Road, Evanston, IL 60208, USA.

[6]Department of Physics and Astronomy, Northwestern University, 2145 Sheridan Road, Evanston, IL 60208, USA.

*To whom correspondence should be addressed.

E-mail: s-stupp@northwestern.edu

Table of Contents

Section 1. **Materials** 3
Section 2. **Synthesis of (1)** 3
Section 3. **Synthesis of (2)** 5
Section 4. **Synthesis of $(NH_4)_2[Mo_3S_{13}]$** 7
Section 5. **Synthesis of Iron Porphyrin Catalyst** 7
Section 6. **Hydrogen Production Experiments** 8
Section 7. **Carbon dioxide Reduction Experiments** 9
Section 8. **Gas Chromatography Traces** 10
Section 9. **Ultraviolet-Visible Absorbance Spectroscopy** 13
Section 10. **Solution Wide and Small Angle x-ray Scattering** 16
Section 11. **Atomic Force Microscopy** 19
Section 12. **Grazing Incidences Wide Angle x-ray Scattering** 21
Section 13. **Scanning Electron Microscopy** 24
Section 14. **Peak matching for unit cell determination** 26
Section 15. **Ultraviolet Photoemission Spectroscopy** 29
Section 16. **Cryogenic Transmission Electron Microscopy** 31
Section 17. **Molecular Dynamics Simulations** 32
Section 18. **Density Functional Theory calculations** 36
Section 19. **^{1}H and ^{13}C NMR Spectra** 37

Materials

Water used for assembly experiments was passed through a Barnstead Nanopure (D3750 hollow fibre filter, 0.2 μm pore size) and UV-irradiated to achieve 18.2 MΩ-cm purity before use. All other chemicals were purchased from Sigma–Aldrich and used without further purification. ^{1}H NMR spectra were acquired using an Agilent DD MR-400 (400 MHz) and ^{13}C NMR spectra acquired using a Bruker AVANCE III (500 MHz, direct cryoprobe). High-resolution mass spectrometry was acquired using an Agilent 6210 LC-TOF (ESI, APCI, APPI).

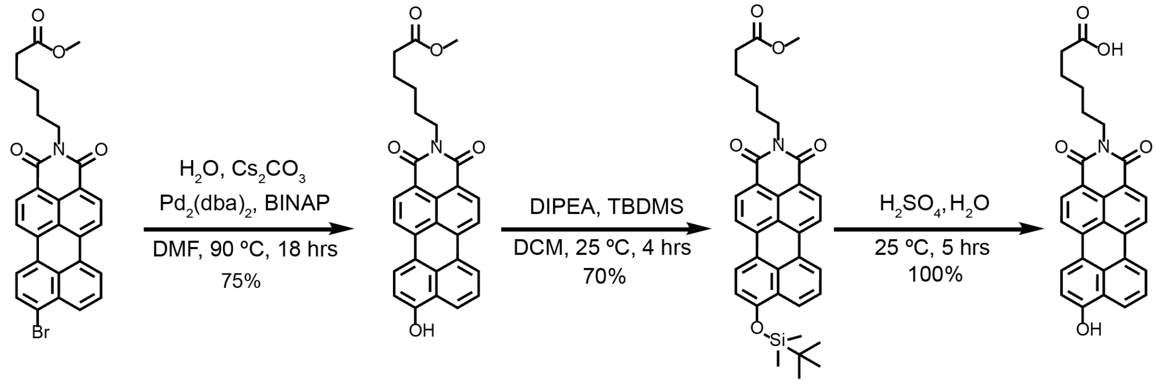

(S1) **(S2)** **(1)**

Scheme 1: Synthesis on 9-hydroxyl-N-(hexanoic acid) perylene-3,4-dicarboximide

9-TBDMS-N-(methyl hexanoate) perylene-3,4-dicarboximide (S2)

A 125 ml Schlenk flask was filled with of **S1** (100 mg, 0.19 mmol)[1], Cs_2CO_3 (430 mg, 1.32 mmol), H_2O (1 ml), DMF (50 ml), and purged with N_2 for 10 minutes. Note the 9-bromo-PMI may not all dissolve until heat is applied. After purging, BINAP (10 mg, 0.016 mmol), and [Pd_2(dba)3] (10 mg, 0.011 mmol) were added and the flask was quickly sealed with a greased glass stopper. The solution was heated at 90 °C for 18 hours after which a green solution was obtained. After cooling the mixture was diluted with 200 ml of 1 M HCl causing a pink/purple precipitate to form. The suspension was filtered with a Buchner funnel and then washed with 500 ml of hot water. The solid was dissolved with a 3:1 DCM/MeOH solution and then dried using a rotary evaporator. The film was then dissolved in 25 ml of DCM and DIPEA (165 μl, 0.95 mmol) was added turning the solution a deep blue color. TBDMS chloride (100 mg, 0.66 mmol) was added

and the solution turned a bright pink color over the next 4 hours. The compound was then purified on a silica column using DCM:MeOH (99.5:0.5), and further purified using recycling gel permeation chromatography using a Japan Analytical Industry Co Lc-9110 Next Series GPC. (Yield 52%).

^{1}H NMR (500 MHz, Chloroform-*d*) δ 8.41 (d, *J* = 8.0 Hz, 1H), 8.39 (d, *J* = 8.1 Hz, 1H), 8.30 (d, *J* = 6.6 Hz, 1H), 8.21 (d, *J* = 8.3 Hz, 1H), 8.18 (d, *J* = 2.8 Hz, 1H), 8.17 (d, *J* = 3.1 Hz, 1H), 8.06 (d, *J* = 8.1 Hz, 1H), 7.54 (t, *J* = 7.7 Hz, 1H), 6.95 (d, *J* = 8.3 Hz, 1H), 4.15 (t, *J* = 7.5 Hz, 2H), 2.35 (t, *J* = 7.5 Hz, 2H), 1.75 (ddq, *J* = 17.4, 15.3, 7.6, 6.9 Hz, 4H), 1.48 (p, *J* = 8.1, 7.4 Hz, 2H), 1.14 (s, 9H), 0.38 (s, 5H).
^{13}C NMR (125 MHz, Chloroform-*d*) δ 174.13, 163.88, 163.86, 154.74, 137.52, 137.26, 131.42, 131.18, 129.75, 129.07, 128.92, 128.24, 126.17, 125.57, 124.99, 124.19, 122.30, 120.46, 119.53, 119.33, 118.58, 114.14, 77.23, 51.49, 40.09, 34.03, 27.75, 26.73, 25.86, 24.73, 18.56, -4.11.
HRMS (ESI-TOF-MS): Expected *m/z*: (579.2437) Observed *m/z*: (579.2441)

9-hydroxyl-N-(hexanoic acid) perylene-3,4-dicarboximide (1)

A scintillation vial was loaded with **S2** (30 mg) and dissolved in 8 ml of H_2SO_4 followed by slow addition of water (2 ml). The solution was left for 5 hours and then precipitated with 100 ml of water, filtered using a Buchner funnel and washed with 500 ml of hot water. The final compound can then be collected off the filter paper with a 3:1 DCM/MeOH solution (sonication may be required). (Yield 100%).

^{1}H NMR (500 MHz, DMSO-d_6) δ 8.66 (d, *J* = 7.5 Hz, 1H), 8.56 (d, *J* = 8.2 Hz, 1H), 8.50 (d, *J* = 8.3 Hz, 1H), 8.36 (m, 3H), 8.26 (d, *J* = 8.3 Hz, 1H), 7.63 (t, *J* = 7.9 Hz, 1H), 7.04 (d, *J* = 8.4 Hz, 1H), 4.02 (t, *J* = 7.6 Hz, 2H), 2.21 (t, *J* = 7.3 Hz, 2H), 1.63 (p, *J* = 7.7 Hz, 2H), 1.55 (p, *J* = 7.5 Hz, 2H), 1.34 (p, *J* = 7.3, 6.6 Hz, 2H).
^{13}C NMR (125 MHz, DMSO-d_6) δ 174.90, 163.51, 163.47, 138.33, 137.64, 131.79, 131.42, 129.91, 128.75, 128.68, 127.58, 126.50, 126.15, 125.97, 125.79, 125.25, 120.50, 120.17, 118.98, 111.12, 79.70, 79.44, 79.17, 33.97, 27.76, 26.56, 24.72.
HRMS (ESI-Ion-Trap-MS): Expected *m/z*: (451.1420) Observed *m/z*: (451.1635)

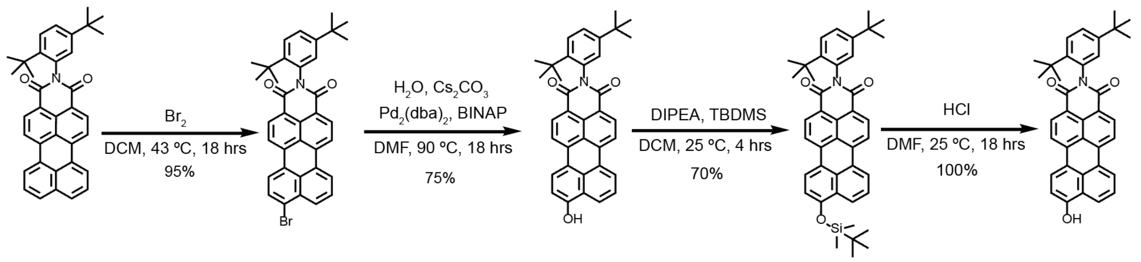

(S3) (S4) (S5) (2)

Scheme 2: Synthesis of 9-hydroxyl-N-(2,5-di-tert-butylaniline) perylene-3,4-dicarboximid.

9-Bromo-N-(2-5-di-tert-butylaniline) perylene-3,4-dicarboximide (S4)

Adapted with changes from reference 1. A solution of **S3** (1.0 g, 1.96 mmol) in CH_2Cl_2 (100 mL) with potassium carbonate (542 mg, 3.92 mmol) in a 250 ml round bottom flask was treated with Br_2 (120 µL, 2.2 mmol) at 25 °C. The flask was attached to a reflux condenser and heated to 43 °C for 18 hours. The mixture was sparged with N_2 to remove excess Br_2, filtered through a cotton plug (to remove residual solid potassium carbonate), and the volatiles were removed on a rotary evaporator. Flash column chromatography (SiO_2, CH_2Cl_2) afforded **S4** (1.1 g, 95%) as a red solid.

^{1}H NMR (500 MHz, Chloroform-*d*) δ 8.60 (d, *J* = 8.0 Hz, 1H), 8.58 (d, *J* = 8.0 Hz, 1H), 8.33 – 8.29 (m, 2H), 8.25 (d, *J* = 8.1 Hz, 1H), 8.19 (d, *J* = 8.4, 1H), 8.05 (d, *J* = 8.2 Hz, 1H), 7.78 (d, *J* = 8.1 Hz, 1H), 7.60 (m, *J* = 2H), 7.47 (dd, *J* = 8.6, 2.3 Hz, 1H), 7.07 (d, *J* = 2.2 Hz, 1H), 1.35 (s, 9H), 1.32 (s, 9H).

^{13}C NMR (125 MHz, Chloroform-*d*) δ 163.73, 148.99, 142.70, 135.53, 135.39, 131.92, 131.67, 130.80, 130.72, 130.02, 128.96, 128.85, 128.36, 127.80, 127.69, 126.92, 126.78, 125.37, 125.16, 125.05, 123.17, 122.49, 120.62, 119.53, 119.26, 34.51, 33.24, 30.73, 30.24.

HRMS (ESI-TOF-MS): Expected *m/z*: (587.1458) Observed *m/z*: (588.1533, M+H)

9-TBDMS-N-(2,5-di-tert-butylaniline) perylene-3,4-dicarboximide (S5)

A 125 ml Schlenk flask was filled with **S4** (100 mg, 0.17 mmol), Cs_2CO_3 (430 mg, 1.32 mmol), H_2O (1 ml), DMF(50 ml), and the mixture was purged with N_2 for 10 minutes. After purging, BINAP (10 mg, 0.016 mmol), and [$Pd_2(dba)_3$] (10 mg, 0.011 mmol) were added and the flask was quickly sealed with a greased glass stopper. The solution was heated at 90 ^{o}C for 4 hours until a green solution was obtained. After cooling the solution was diluted with 200 ml of 1 M HCL causing a pink/purple precipitate to form. The suspension was filtered with a Buchner funnel and then washed

with 500 ml of hot water. The solid was dissolved with a 3:1 DCM/MeOH solution and then dried using a rotary evaporator. The film was then dissolved in 25 ml of DCM (dry) and DIPEA (165 µl, 0.95 mmol) was added turning the solution a deep blue color. TBDMS chloride (100 mg, 0.66 mmol) was added and the solution turned a bright pink color over the next 4 hours. The compound was then purified on a silica column using 3:1 hexane/DCM mixture, and further purified using recycling gel permeation chromatography. (Yield 52%).

^{1}H NMR (500 MHz, Chloroform-*d*) δ 8.61 (d, *J* = 8.0 Hz, 1H), 8.58 (d, *J* = 8.0 Hz, 1H), 8.44 (d, *J* = 6.7 Hz, 1H), 8.37 (d, *J* = 8.1 Hz, 1H), 8.30 (d, *J* = 8.4 Hz, 1H), 8.27 (d, *J* = 8.3 Hz, 1H), 8.24 (d, *J* = 8.1 Hz, 1H), 7.61 (t, *J* = 7.7 Hz, 1H), 7.59 (d, *J* = 8.6 Hz, 1H), 7.45 (dd, *J* = 8.6, 2.3 Hz, 1H), 7.04 (d, *J* = 2.2 Hz, 1H), 7.00 (d, *J* = 8.3 Hz, 1H), 1.34 (s, 9H), 1.31 (s, 9H), 1.14 (s, 9H), 0.39 (d, *J* = 2.7 Hz, 6H).
^{13}C NMR (125 MHz, Chloroform-*d*) δ 164.02, 153.82, 148.89, 142.75, 137.01, 136.75, 132.20, 131.00, 130.76, 128.07, 127.63, 127.32, 126.82, 125.25, 125.01, 124.66, 124.15, 123.35, 121.40, 119.99, 118.85, 118.70, 117.74, 113.16, 34.49, 33.21, 30.71, 30.23, 24.81, 17.51, -5.13.
HRMS (ESI-TOF-MS): Expected *m/z*: (639.3174) Observed *m/z*: (639.3169)

9-hydroxyl-N-(2,5-di-tert-butylaniline) perylene-3,4-dicarboximide (2)

A 100 ml round bottom flask was loaded with **S5 (**60 mg), and dissolved in 30 ml of DMF, 5 ml of 2 M HCl was added and the solution was stirred overnight. The compound was then precipitated with 100 ml 2 M HCl, filtered, and washed with 500 ml of hot water. The final compound can then be collected off the filter paper with a 3:1 DCM/MeOH solution (sonication may be required). (Yield 100%).

^{1}H NMR (500 MHz, DMSO-d_6) δ 11.27 (s, 1H), 8.74 (d, *J* = 6.6 Hz, 1H), 8.67 (d, *J* = 8.2 Hz, 1H), 8.59 (d, *J* = 8.5 Hz, 1H), 8.49 (d, *J* = 8.3 Hz, 1H), 8.46 (d, *J* = 8.1 Hz, 1H), 8.42 (d, *J* = 8.1 Hz, 1H), 8.30 (d, *J* = 8.3 Hz, 1H), 7.69 (t, *J* = 7.9 Hz, 1H), 7.53 (d, *J* = 8.6 Hz, 1H), 7.41 (dd, *J* = 8.6, 2.3 Hz, 1H), 7.15 (d, *J* = 2.3 Hz, 1H), 7.11 (d, *J* = 8.4 Hz, 1H), 1.26 (s, 9H), 1.18 (s, 9H).
^{13}C NMR (125 MHz, DMSO-d_6) δ 164.78, 157.68, 149.75, 143.82, 138.65, 137.98, 134.13, 132.06, 131.71, 130.52, 128.87, 128.79, 128.70, 128.54, 127.70, 126.68, 126.44, 126.07, 125.97, 125.80, 125.24, 120.87, 120.73, 120.04, 119.23, 119.15, 111.10, 35.60, 34.43, 31.83, 31.51.
HRMS (ESI-TOF-MS): Expected *m/z*: (525.2303) Observed *m/z*: (525.2304)

***$(NH_4)_2[Mo_3S_{13}]$*[2]**

The procedure from Kibsgaard *et al.*[2] was modified as follows: Ammonium sulfide solution, 20 wt% in H_2O, (7.692 mL) was treated with elemental sulfur (S_8, 0.5 g, 15.6 mmol). The mixture was agitated and sonicated until all of the sulfur dissolved, yielding a bright orange solution. In a separate vial, $(NH_4)_6Mo_7O_{24}\cdot 4\ H_2O$ (1 g, 0.81 mmol) was dissolved in water (5.1 mL) and this solution was added to the sulfur solution resulting in a dark red solution. The flask containing this mixture was sealed and heated at 95°C for 5 d. Note: make sure the flask is properly sealed, evaporation of water during heating will result in formation of impurities. After 5 d, the dark red crystals were filtered, washed with water and ethanol, and then transferred to a flask charged with toluene (100 mL). The toluene was heated to boil in order to dissolve away any unreacted sulfur. The solution was decanted and the solid was washed repeatedly with fresh toluene until no more sulfur was present (3 washes). The crystals were then filtered and dried under vacuum to give $(NH_4)_2[Mo_3S_{13}]$. Crystals were stored under Ar in a –20 °C freezer until use. To make a catalyst solution in water, $(NH_4)_2[Mo_3S_{13}]$ (2 mg) dissolved in water (15.06 mL). The solution was heated, vortexed, and sonicated repeatedly to get yield a yellow solution.

Iron(III) 5,10,15,20-Tetra(4'-N,N,N-Trimethylanilinium) Porphyrin Pentachloride

5,10,15,20-Tetra(4'-N,N,N-trimethylanilinium) porphyrin tetrachloride (33 mg, 0.0338 mmol, Frontier Scientific), anhydrous iron(II) bromide (130 mg, 0.604 mmol, Alfa-Aesar), and 2,6-lutidine (39 µL, 0.338 mmol, Sigma-Aldrich) were dissolved in 60 mL dry methanol in a 250 ml round bottom flask. The solution was degassed with nitrogen for 10 minutes and then refluxed under nitrogen for 7 days. The methanol was removed by rotary evaporation and the resulting solid residue was sonicated in THF (125 mL) and filtered. The solid collected was washed with dichloromethane and then dissolved in methanol. This solution was concentrated by rotary evaporation, then acidic THF was added to precipitate the pentachloride iron(III) product.

H_2 Production Experiments

Solutions of **1** (100 μL, 7.25 mM) were first gelled with poly(diallyl dimethyl ammonium) chloride (PDDA) (20 μL, 300 mM) in a VWR Microwave Vial 0.5-2 mL (Catalog Number 89079-406) and allowed to age overnight. Ascorbic acid solutions (1.7 M) were adjusted to pH 4 using 4 M NaOH (measured using a Fisher Scientific Accumet Research AR50 Dual Channel pH/Ion/Conductivity Meter, calibrated with pH 4.0 and pH 7.0 standard solutions. 20 μL of the $(NH_4)_2[Mo_3S_{13}]$ solution (180 μM) or 20 μL of (288 μM) Fe-porphyrin was then added to the gels, along with 700 μL of ascorbic acid solution as a source of protons and sacrificial electrons. Vials were sealed with a septum, further tightened with zip-ties, and wrapped in Parafilm to ensure proper sealing. After purging for 10 minutes with Ar, the samples were illuminated for 18 hours with a Schott DCR III lamp equipped with 150 W EKE bulb and fiber optic goosenecks. Samples were placed approximately 1.5 cm from the fiber optic light source (power output ~250 W/cm^2). For H_2 identification and quantification, a 300 μL aliquot was taken from the sample vial (7.7 mL headspace) and injected onto a gas chromatograph (Shimadzu GC-2014) equipped with a 5 Å molecular sieve column, Ar carrier gas, and a thermal conductivity detector. Eight-point calibration curves for H_2 and N_2 were created using a standard (7% H_2 balanced with N_2) and integrated peak areas were used to determine the H_2 concentration in the sample headspace at STP.

CO_2 reduction experiments

Solutions of **1'** (100 μL, 8.7 mM) were added to a VWR Microwave Vial 0.5-2 mL (Catalog Number 89079-406). A sacrificial electron donor (sodium ascorbate) and catalyst (Fe-porphyrin) solution was added to each vial so the final solution volume was 1 ml. The ascorbate concentration was 0.5 M and the catalyst concentration was 6 μM. Vials were sealed with a septum, further tightened with zip-ties, and wrapped in Parafilm wax and degassed with CO_2 for 10 minutes. After degassing 100 μl of 4 M NaOH was added. Samples were illuminated for 48 hours with a Schott DCR III lamp equipped with 150 W EKE bulb and fiber optic goosenecks. Samples were placed approximately 1.5 cm from the fiber optic light source (power output ~250 W/cm^2). For CO identification and quantification, a 300 μL aliquot was taken from the sample vial (7.7 mL headspace) and injected onto a gas chromatograph (Shimadzu GC-2014) equipped with a 5 Å molecular sieve column, Ar carrier gas, and a thermal conductivity detector. Eight-point calibration curves for CO and N_2 were created using a standard (5% CO balanced with N_2) and integrated peak areas were used to determine the CO concentration in the sample headspace at STP. Peak areas are only estimated due to the small CO peak intensity which was not fully resolved from the N_2 peak.

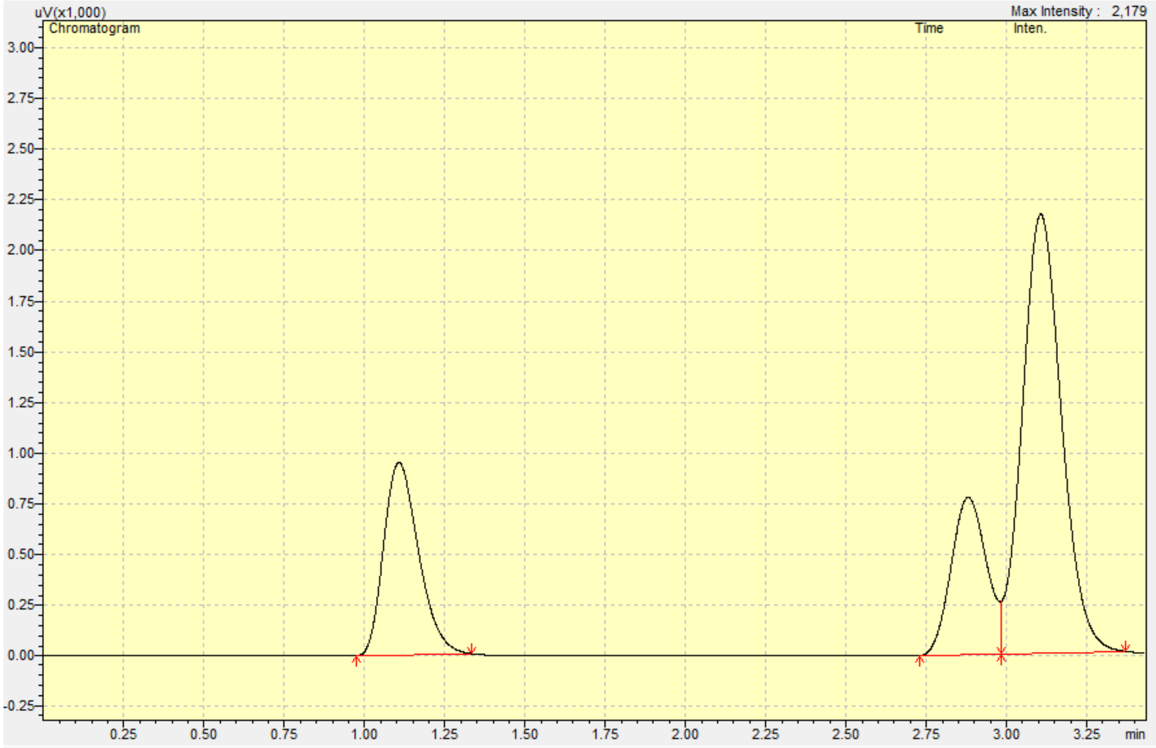

SI Figure 1: Example GC trace for photocatalytic hydrogen production experiment using **1** with $(NH_4)_2[Mo_3S_{13}]$ as the catalyst.

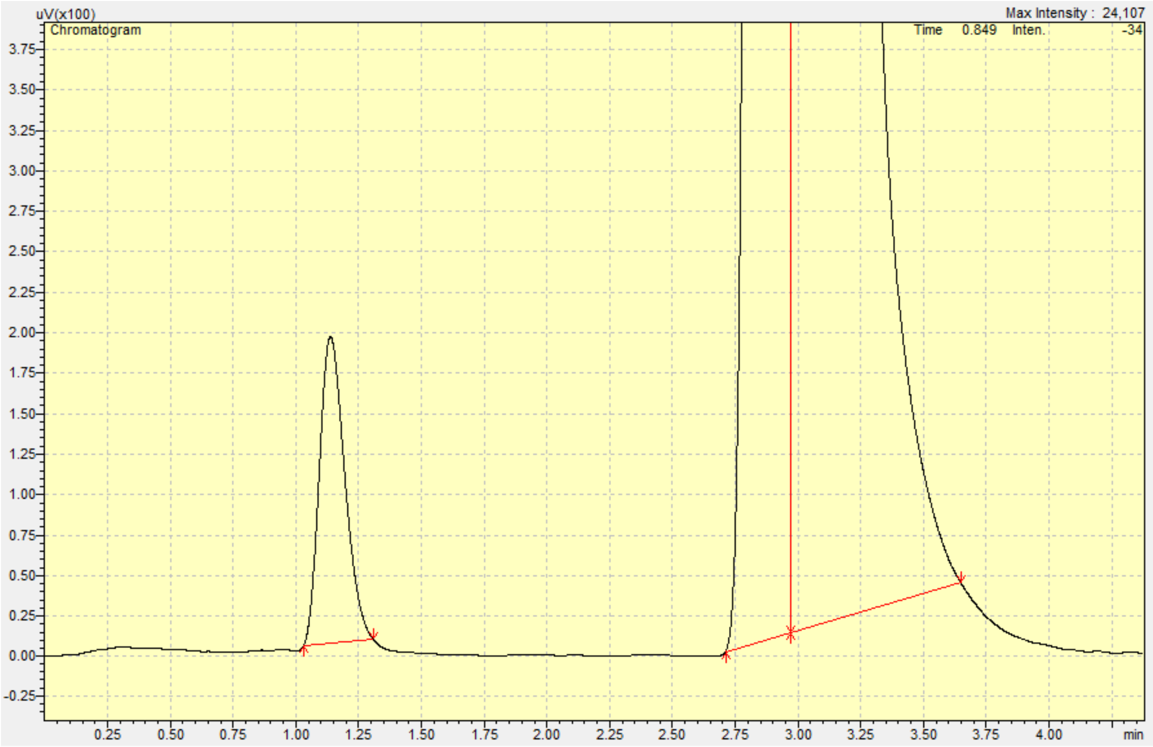

SI Figure 2: Example GC trace for photocatalytic hydrogen production experiment using **1'** with Fe-p-TMA as the catalyst.

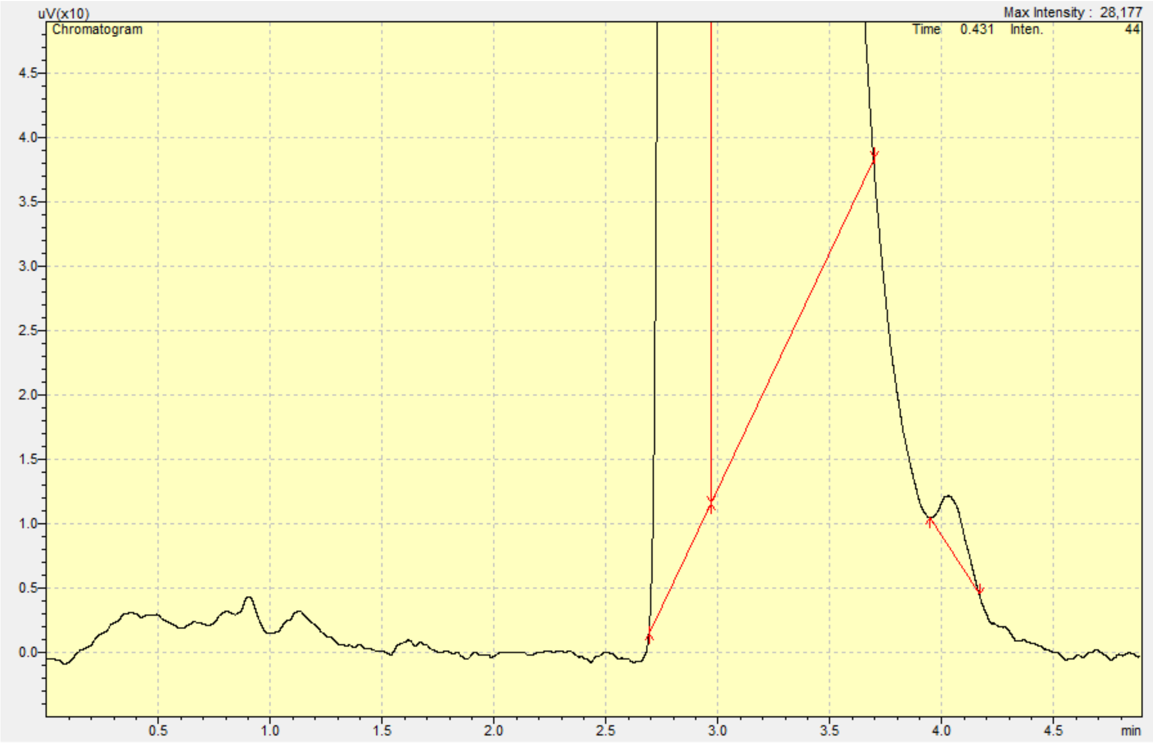

SI Figure 3: Example GC trace for photocatalytic CO_2 reduction experiment using **1'** with Fe-p-TMA as the catalyst. TON are only estimated due to the small CO peak area which was not fully resolved from the N_2 peak.

Ultraviolet – Visible Absorbance Spectroscopy

Absorbance spectroscopy of **1** and **2** was performed using either a 0.05 mm path length, closed demountable quartz spectrophotometer cell (Starna Cells) or a 1 mm path length cuvette on a Shimadzu UV-1800 UV Spectrophotometer. For pH titrations a Thermo Fisher Nano-drop 1000 was used to collect absorbance data.

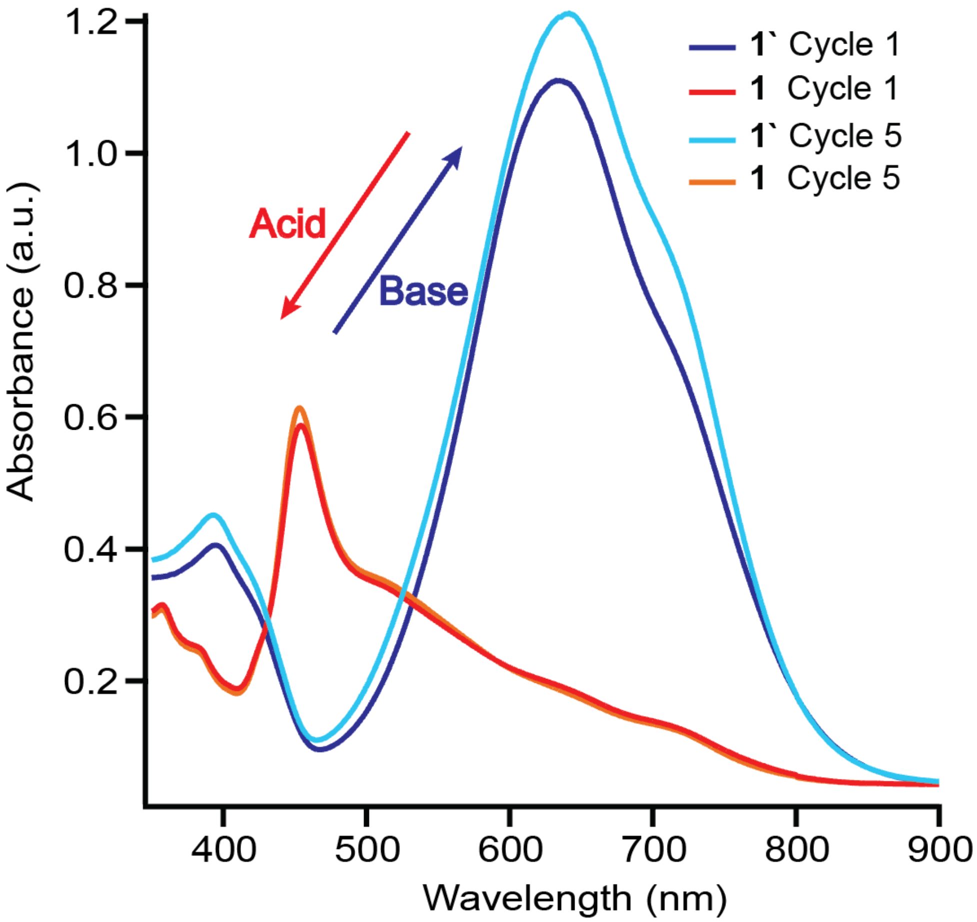

SI Figure 4: UV-Vis Absorbance of **1** (8.7 mM) during acid/base cycling. A fresh PMI film was dissolved in water with 2 eq of NaOH (**1'** cycle 1) followed by addition of 1 eq of HCl (**1** cycle 1). Then 1 eq of NaOH and HCl were added 4 additional times and the absorbance spectra were recorded at the end of cycling.

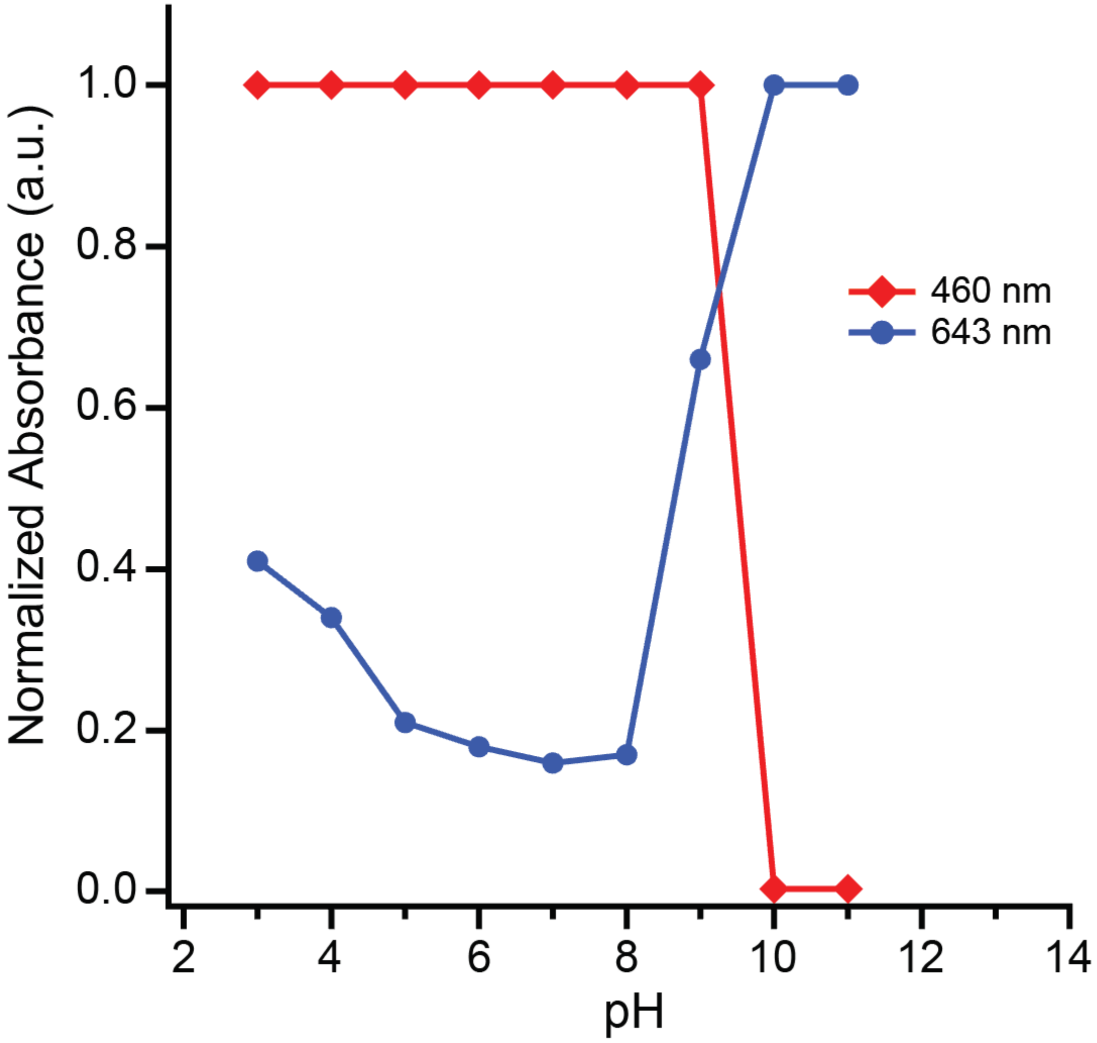

SI Figure 5: UV-Vis titration of **1** incubated in buffers of various pH. Solutions of **1** (8.7 mM) were diluted tenfold into buffers ranging from pH 3-11 and allowed to incubate for 10 minutes. The pK_a was determined to be around pH 9 where the normalized absorbance of the peak at 460 nm (protonated) and 643 nm (deprotonated) are nearly equal. Normalization is done by setting the largest absorbance to 1 for each spectrum. Note for pH 3-9 the largest absorbance occurs at 460 nm, and at pH 10 and above the largest absorbance occurs at 643 nm.

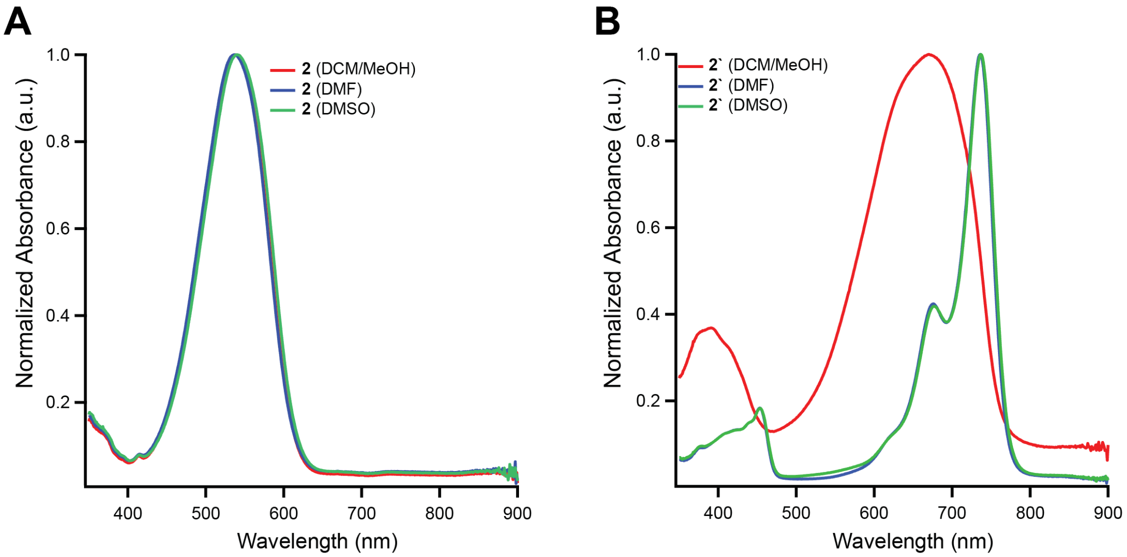

SI Figure 6: Normalized UV-vis absorbance spectra of A) **2** and B) **2'** at 0.87 mM. For compound **2,** samples were dissolved in the appropriate solvent and 10 ul of 4 M HCl was added. Samples were then vortexed and sonicated to ensure complete protonation. For compound **2'**, the same method was used replacing the HCl with NaOH. Solvatochromic behavior under basic conditions arises from the solvent's ability to stabilize different ground state resonance structures of **2'**.

Wide Angle X-ray Scattering (WAXS)/ Small Angle X-Ray Scattering (SAXS)

X-ray measurements were performed using beam line 5ID-D, in the DuPont-Northwestern-Dow Collaborative Access team (DND-CAT) Synchrotron Research Center at the Advanced Photon Source, Argonne National Laboratory. X-ray energy of 12 keV corresponding to a wavelength of 1.0332 $Å^{-1}$ was selected using a double-crystal monochromator. Data was collected simultaneously across three Rayonix detectors, model numbers SAXS – LX170HS, MAXS - LX170HS, WAXS – MX170HS. Sample to detector distances were as follows: 200.82 mm for SAXS, 1013.4 mm for MAXS, and 8500 mm for WAXS. The scattering intensity was recorded in the interval $0.001687 < q < 3.147$ $Å^{-1}$. The wave vector defined as

$$q = (4\pi/ \lambda) \sin(\theta/2)$$

where θ is the scattering angle. Exposure time was 1 second. Samples were oscillated with a syringe pump during exposure to prevent beam damage. Background samples containing water or 50 mM NaCl were also collected in order to perform background subtractions.

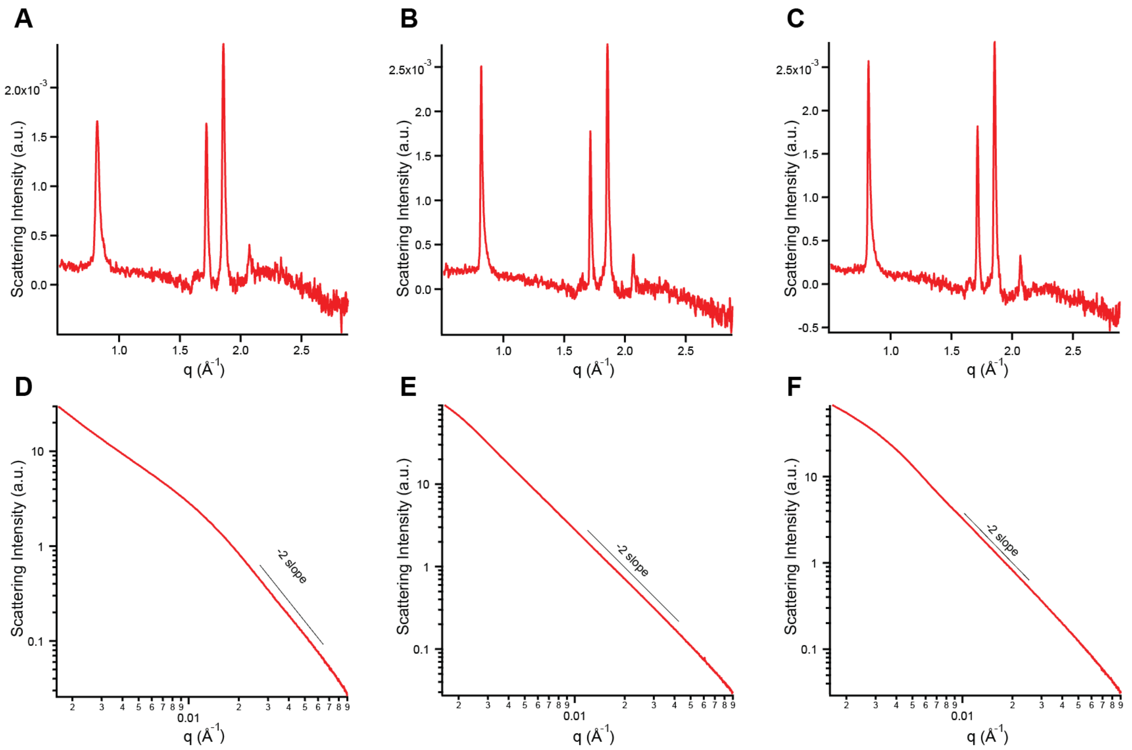

SI Figure 7: A) Solution WAXS of freshly prepared **1**. B) Solution WAXS of **1** after annealing at 80°C. C) Solution WAXS of **1** after annealing in a 50 mM NaCl solution at 80°C. D) Solution SAXS of freshly prepared **1.** E) Solution SAXS of **1** after annealing at 80°C. F) Solution SAXS of **1** after annealing in a 50 mM NaCl solution at 80°C. All samples have PMI concentration of 7.25 mM

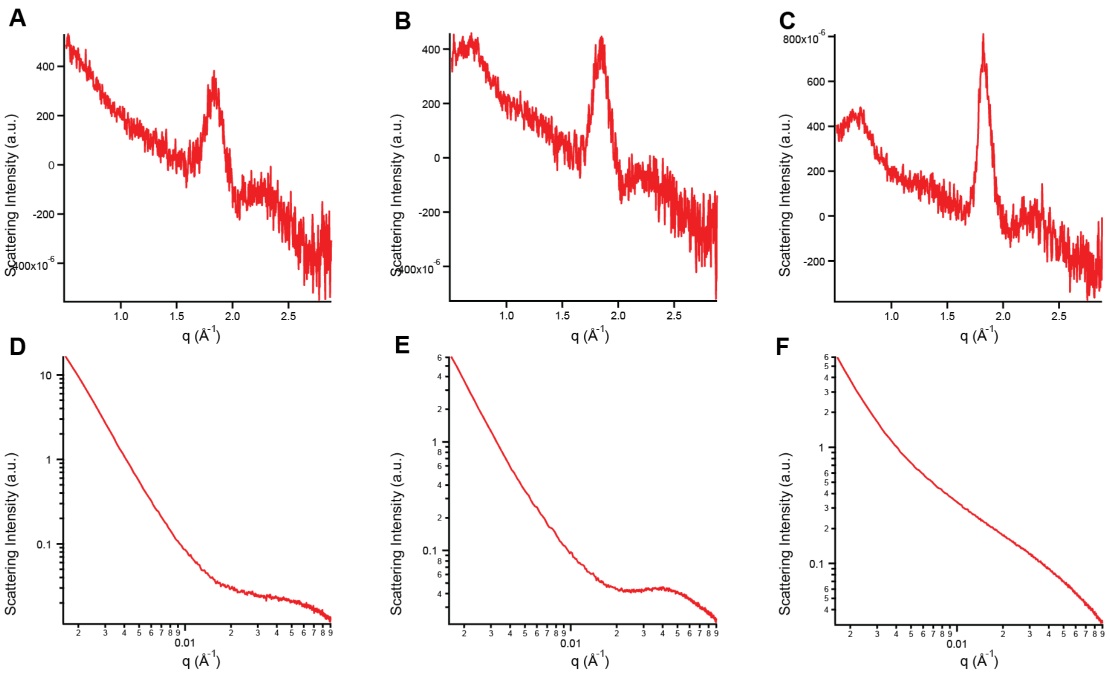

SI Figure 8: A) Solution WAXS of freshly prepared **1'**. B) Solution WAXS of **1'** after annealing at 80°C. C) Solution WAXS of **1'** after annealing in a 50 mM NaCl solution at 80°C. D) Solution SAXS of freshly prepared **1'**. E) Solution SAXS of **1'** after annealing at 80°C. F) Solution SAXS of **1'** after annealing in a 50 mM NaCl solution at 80°C. All samples have PMI concentration of 7.25 mM

Atomic force microscopy (AFM)

AFM characterization was performed using a Bruker Dimension ICON atomic force microscope (Bruker Co.) at ambient conditions. Tapping mode was utilized with single-beam silicon cantilevers with a nominal oscillation frequency of 300 kHz. Solutions of 7.25 mM PMI were spin coated (10 µL) on pre-cleaned silicon substrates at 2000 rpm. Substrates were cleaned by sonicating in isopropyl alcohol for 30 minutes.

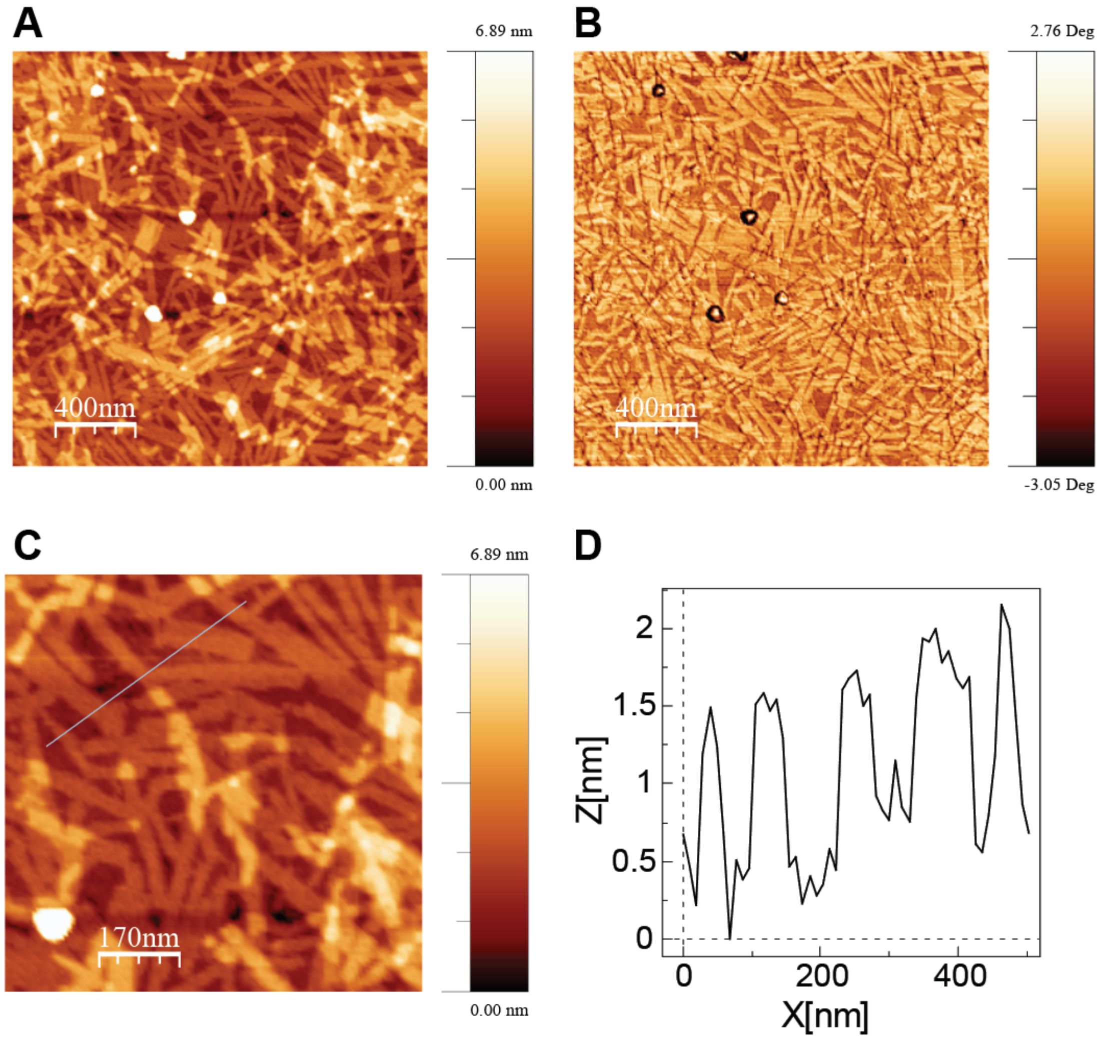

SI Figure 9: AFM images of **1**. A) Height profile image. B) Phase contrast image. C) Higher magnification of image A with a line cut for height measurement. D) Line cut measurements from image C.

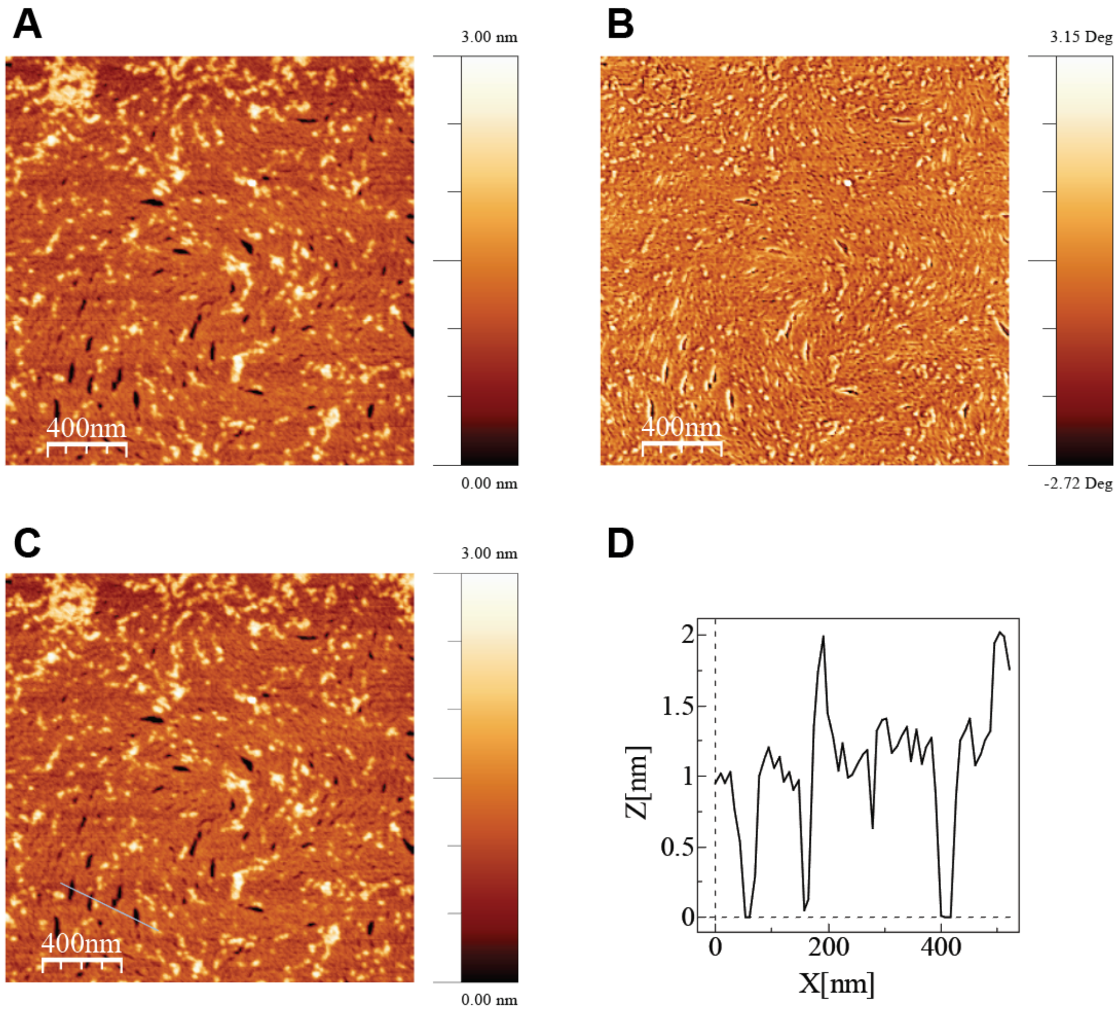

SI Figure 10: AFM Images of **1'**. A) Height profile image. B) Phase contrast image. C) Higher magnification image A with a line cut for height measurement. D) Line cut measurements from image C.

Grazing Incidence Wide Angle X-ray Scattering (GIWAXS)

GIWAXS data was collected at Argonne National Laboratory's Advanced Photon Source, in sector 8-ID-E. The beam energy was 7.35 keV, sample-to-detector distance was 212 mm, and the incident angle was 0.2°. Samples were prepared by drop casting 30 μL of PMI (7.25 mM) solution or gel onto a clean glass substrate. The solutions were allowed to dry in air overnight. X-rays were detected using a Pilatus 1M detector. 2D data and line cuts were processed using GIXSGUI, a Matlab toolbox developed by Zhang Jiang at Sector 8 of the APS for processing GIWAXS data.[3]

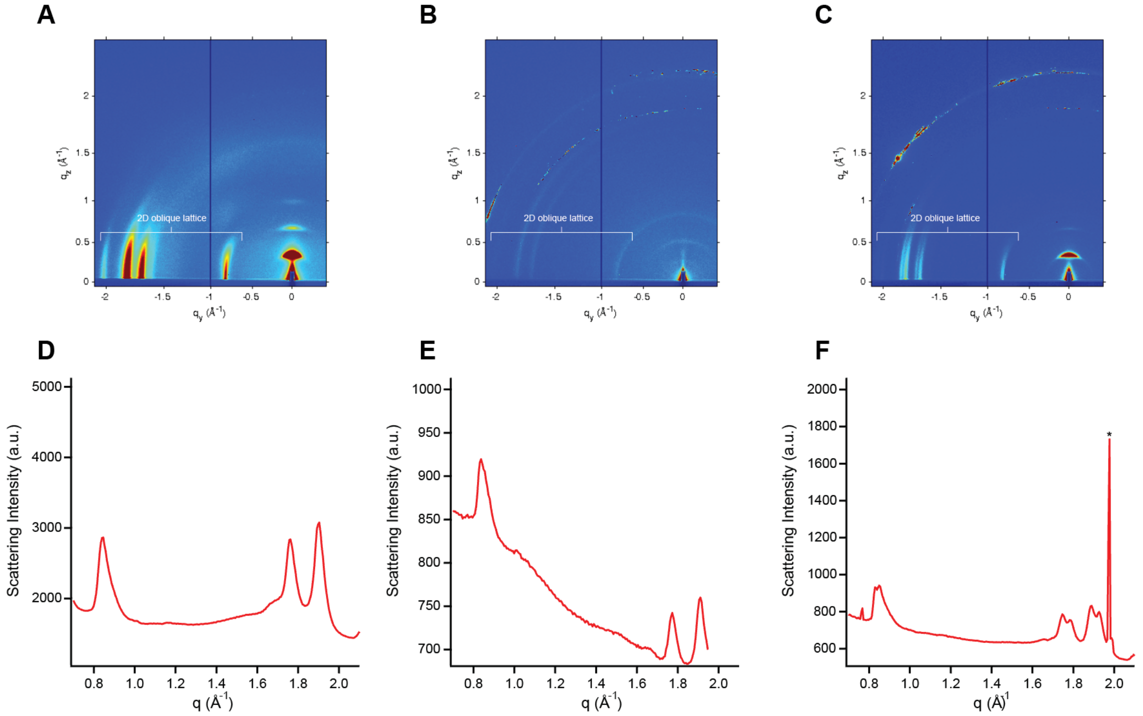

SI Figure 11: Grazing incidence wide-angle x-ray scattering (GIWAXS) data for **1** and the corresponding line cuts. A) Freshly prepared **1**. B) **1** sample gelled with 50 mM $CaCl_2$. C) **1** annealed in a 50 mM NaCl solution. * indicates a peak form NaCl and does not correspond to the 2D PMI lattice. D) Line cut of the data in panel A. E) Line cut of the data in panel B. F) Line cut of the data in panel C. PMI samples were drop cast at 7.25 mM.

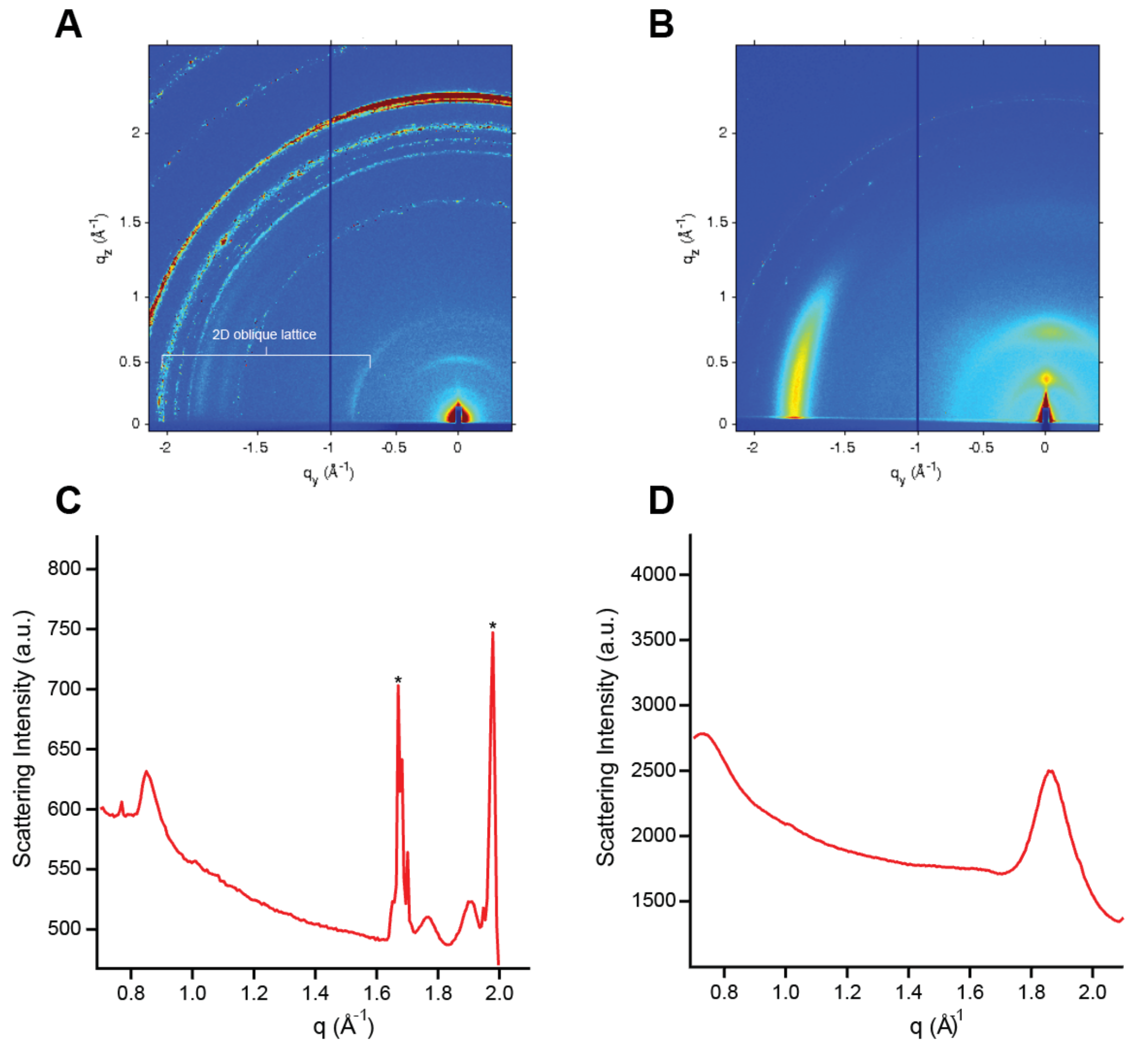

SI Figure 12: Grazing incidence wide-angle x-ray scattering (GIWAXS) data for **1'** and the corresponding line cuts. A) **1'** sample gelled with 50 mM $CaCl_2$. * indicate the $CaCl_2$ peaks that are present due to excess salt on the area where this data was collected. These peaks are not related to the 2D PMI lattice. B) **1'** annealed in a 50 mM NaCl solution. C) Line cut of the data in panel A. D) Line cut of the data in panel B. PMI samples were drop cast at 7.25 mM.

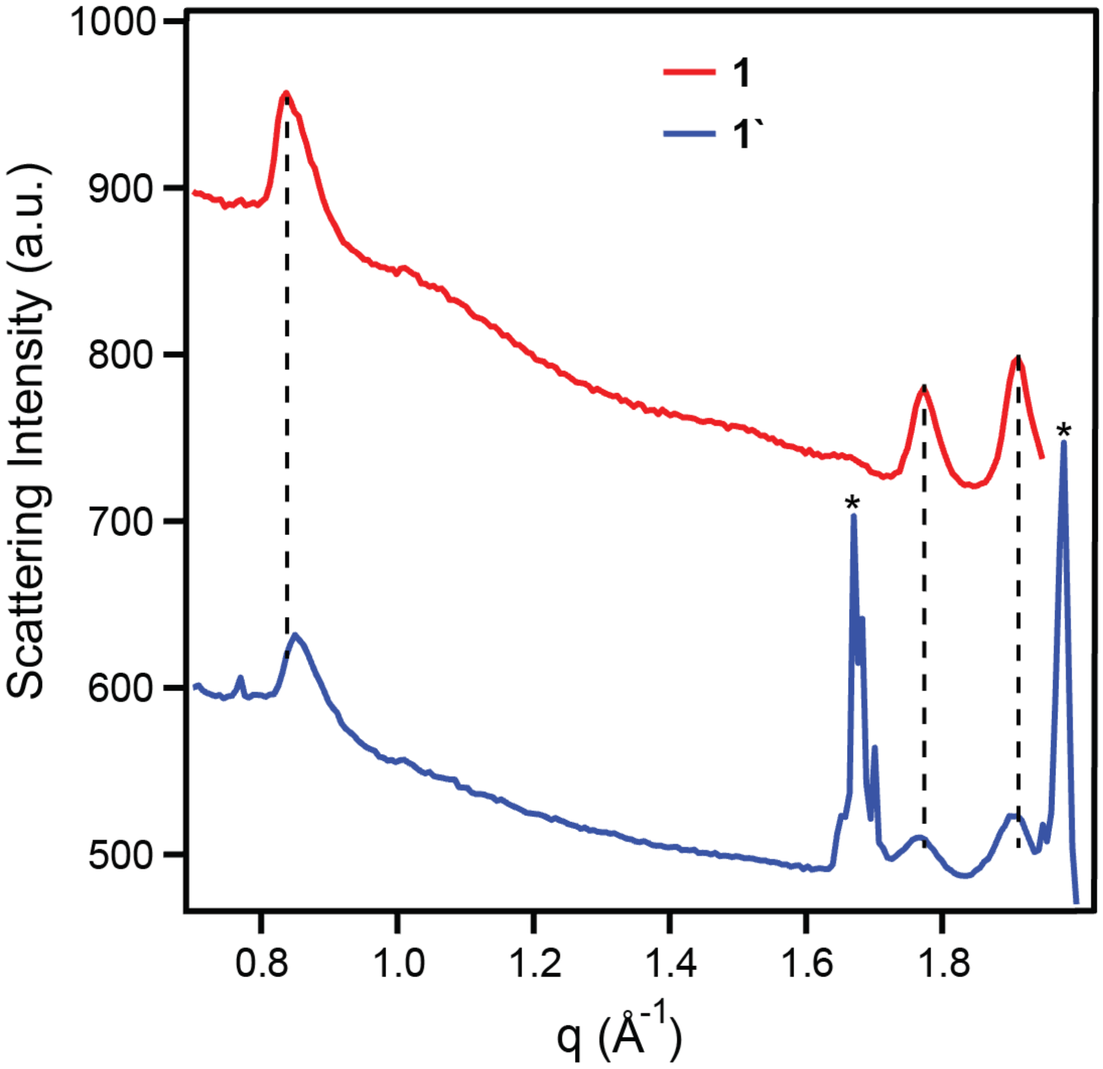

SI Figure 13: GIWAXS line cuts for **1** and **1'** samples (7.25 mM) gelled with 43 mM $CaCl_2$ then drop cast onto glass slides. * indicate the $CaCl_2$ peaks that are present due to excess salt on the area where this data was collected. These peaks are not related to the 2D PMI lattice.

Scanning Electron Microscopy (SEM)

SEM images of were collected at the EPIC imaging facility using a Hitachi SU8030 microscope. Samples of **1** and **1'** (100 µl, 8.7 mM) were placed in a conical vial and $CaCl_2$ (20 µl, 300 mM) was added without agitation. After 18 hours the samples were placed in a 30% ethanol solution for 10 minutes. Every 10 minutes the samples were placed in ethanol solution of increasing concentration (10% steps). Samples were then dried using a Tousimis samdri-795 critical point dryer, coated with 8 nm of osmium, and imaged.

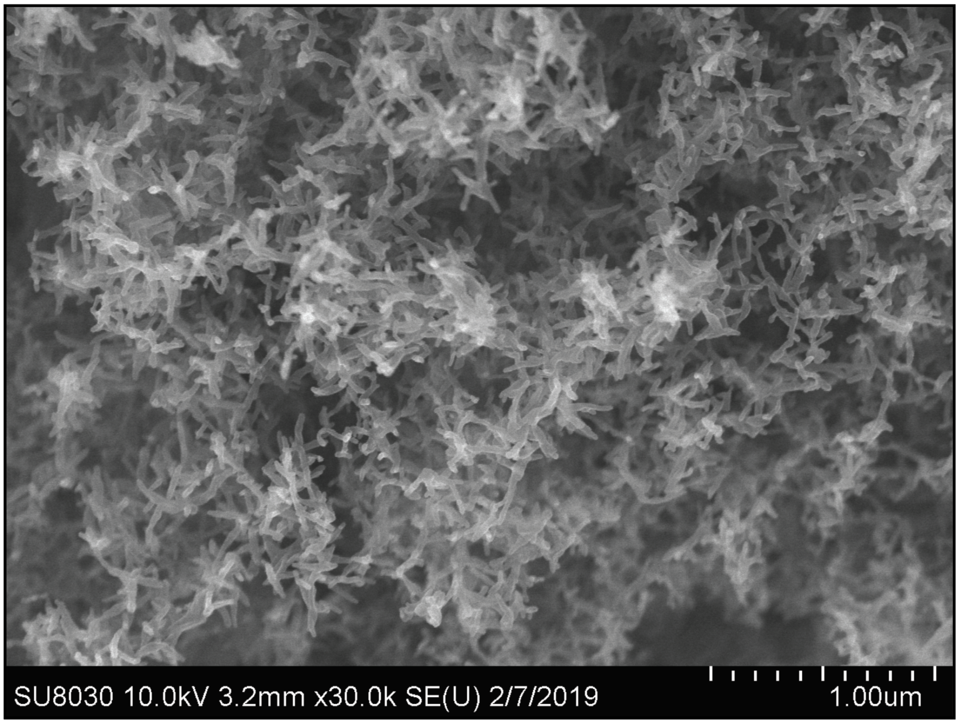

SI Figure 14: SEM image of **1** (100 µl, 8.7 mM) gelled with $CaCl_2$ (20 µl, 300 mM).

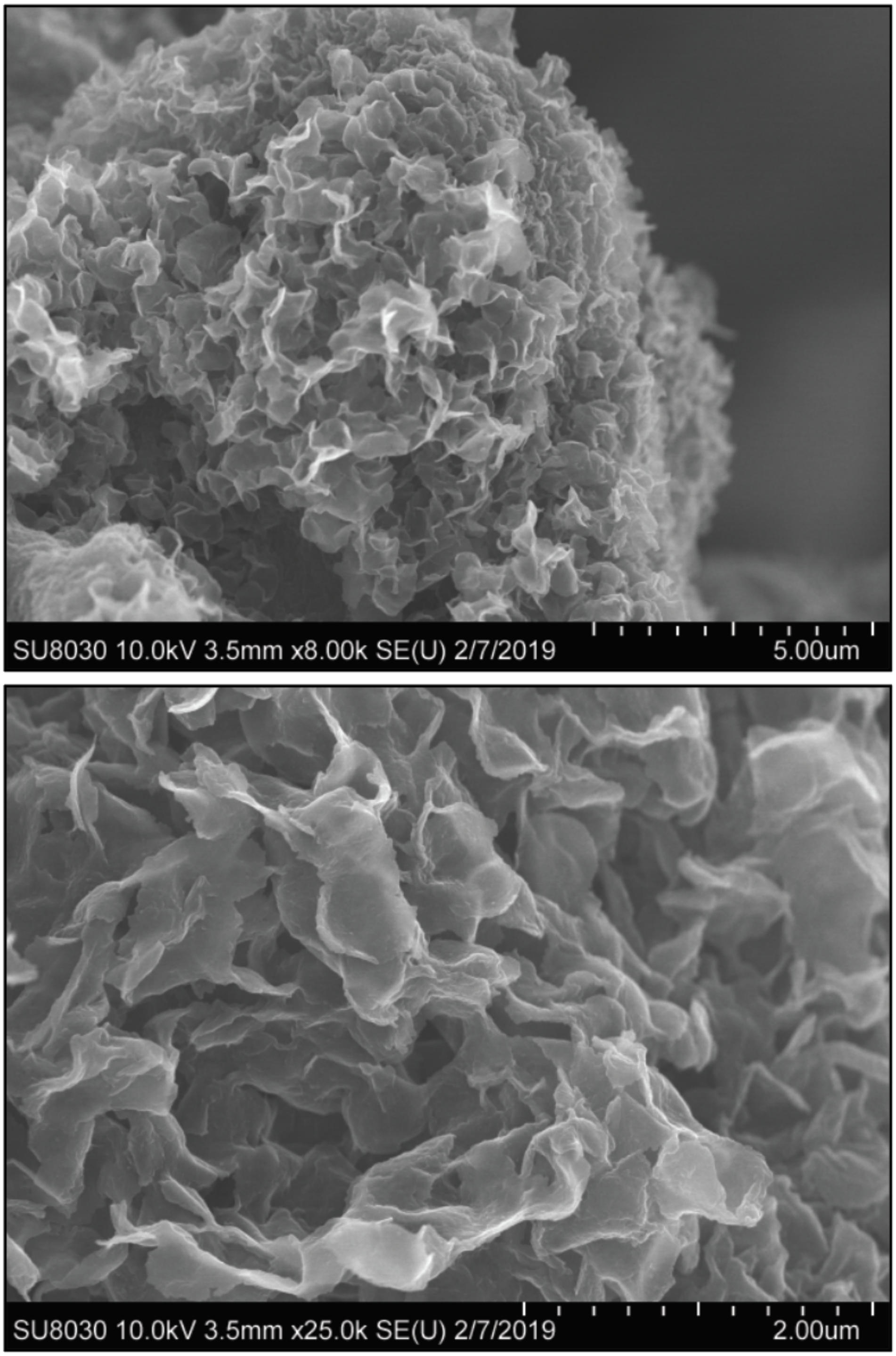

SI Figure 15: SEM image of **1'** (100 µl, 8.7 mM) gelled with $CaCl_2$ (20 µl, 300 mM).

Peak matching for unit cell determination

Peak positions were used to deduce the lattice parameters[4]. The inferred unit cell parameters are: a = 16.6Å, b = 3.7 Å and γ = 64.6°. Molecular sizes of perylene suggest that the basis consists of two perylene molecules. Each perylene molecule was modelled as a uniform slab with a rod attached to the top side of the slab (representing respectively the fused aromatic rings and pentadecyl chain). Slabs were oriented with their longest dimension along the z-axis, perpendicular to the plane of the sheet, and with a free rotational angle about this axis. Form factor of the two-molecule basis was constructed with rotational angles and slab width as parameters in the model. Domain sizes were deduce through Scherrer formula on the (01) peak for **(1)** and on the sole peak for **(1`)**.

Line-cut of the GIWAXS 2D image at $q_z \approx 0$ Å^{-1} was taken and background subtracted. By assuming nanosheets lying flat on the substrate surface, the intensity variations of the peaks are due to the projection of form factor on the sheet, i.e. when $q_z = 0$. The parameterized formula for the projected form factor was fitted to the line-cut. Deduced rotational angles are shown on Fig SI11.

The WAXS scattering pattern was simulated by the scattering theory for randomly oriented 2D structures in solution which accounts for asymmetric line-shape of the peaks. This model-based scattered intensity was fitted by the Levenberg–Marquardt algorithm to the background subtracted WAXS experimental data to extract the rotational angles best reproducing the relative intensities and line shapes of the peaks. Obtained angles are shown on Fig SI12.

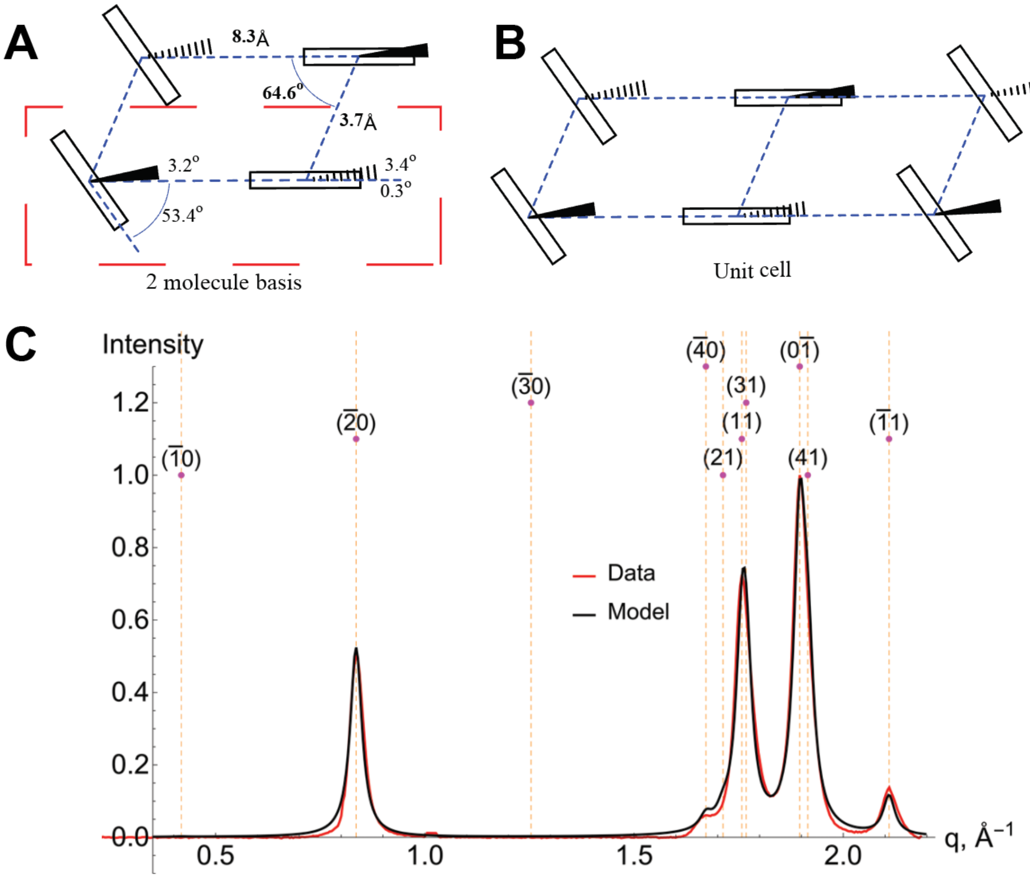

SI Figure 16: Unit cell determination of compound **1** using GIWAXS data. A) Simplified basis set showing two molecules which full describe the molecules locations and rotation angles on the 2D oblique lattice. Rectangles represent the PMI core while wedge and hatches represent the carboxyl pentyl chains above and below the PMI stable. B) Complete unit cell. C) GIWAXS line cut and fitted model.

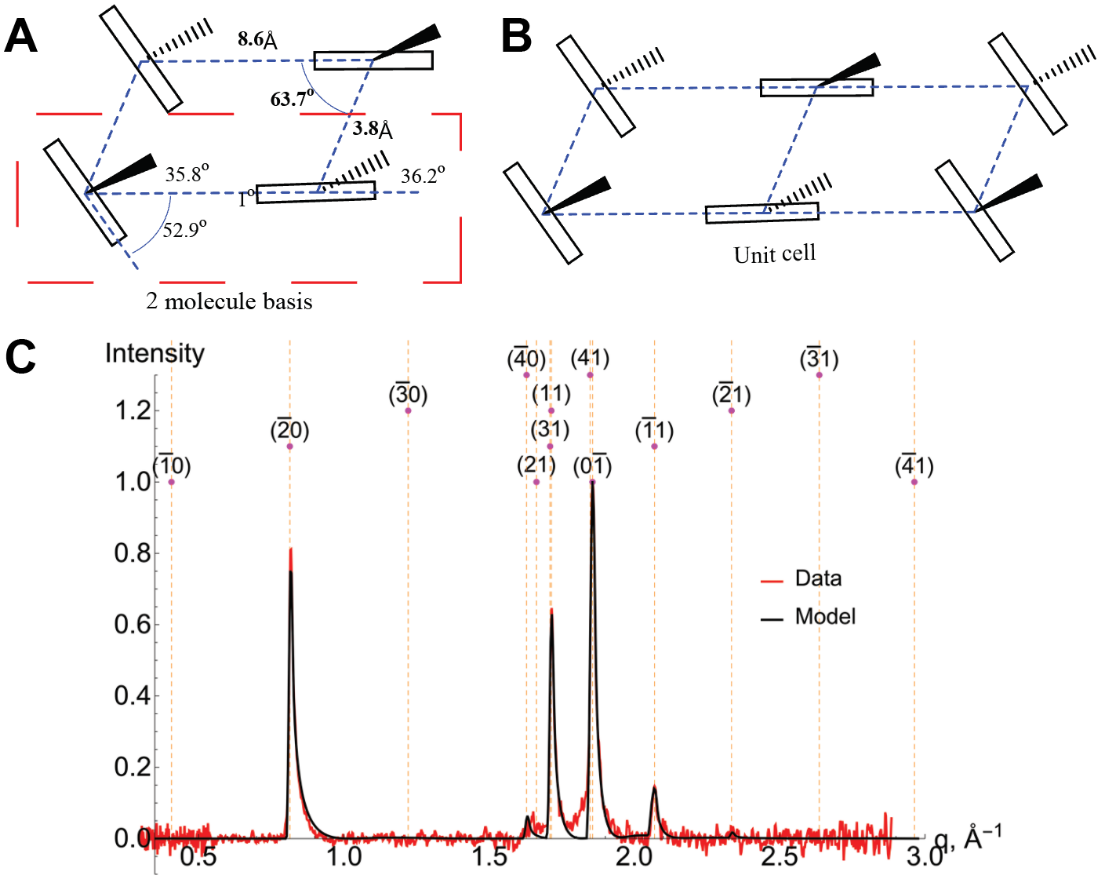

SI Figure 17: Unit cell determination of compound **1** using solution WAXS data. A) Simplified basis set showing two molecules which full describe the molecules locations and rotation angles on the 2D oblique lattice. Rectangles represent the PMI core while wedge and hatches represent the carboxyl pentyl chains above and below the PMI stable. B) Complete unit cell. C) Solution WAXS scattering data and fitted model.

Ultraviolet Photoemission Spectroscopy

Ultraviolet Photoelectron Spectroscopy (UPS) data was used to determine the HOMO level for all molecules in monomeric and assembled states. Samples were prepared by drop-casting 25 μL of 7.25 mM PMI from water and from chloroform/methanol mixtures for assembled and unassembled states, respectively. These samples were drop-cast onto conductive indium tin oxide (ITO) substrates which were affixed to the UPS sample holder with conductive carbon tape. UPS data was collected using a Thermo Scientific ESCALAB 250Xi with a -5.0 V sample bias and a He I ($h\nu$ = 21.2 eV) source. The spectrum width of UPS data is related to the work function, Φ, according to the equation $\Phi = h\nu - BE$, where BE is the binding energy of the electron in the HOMO. The edges of the spectrum were fit in order to determine the binding energy.[12] In the inelastic cutoff region, the intercept between the tangent to the main peak and a linear fit to the background level was used as the edge of the spectrum. At the Fermi edge, the onset of the peak was determined as energy at which the intersection between the local tangent to the spectrum and a linear fit to the background level occurs. The difference in energy between the Fermi edge and the inelastic cutoff region was then subtracted from the incident energy (21.2 eV) in order to determine the HOMO level of the material.

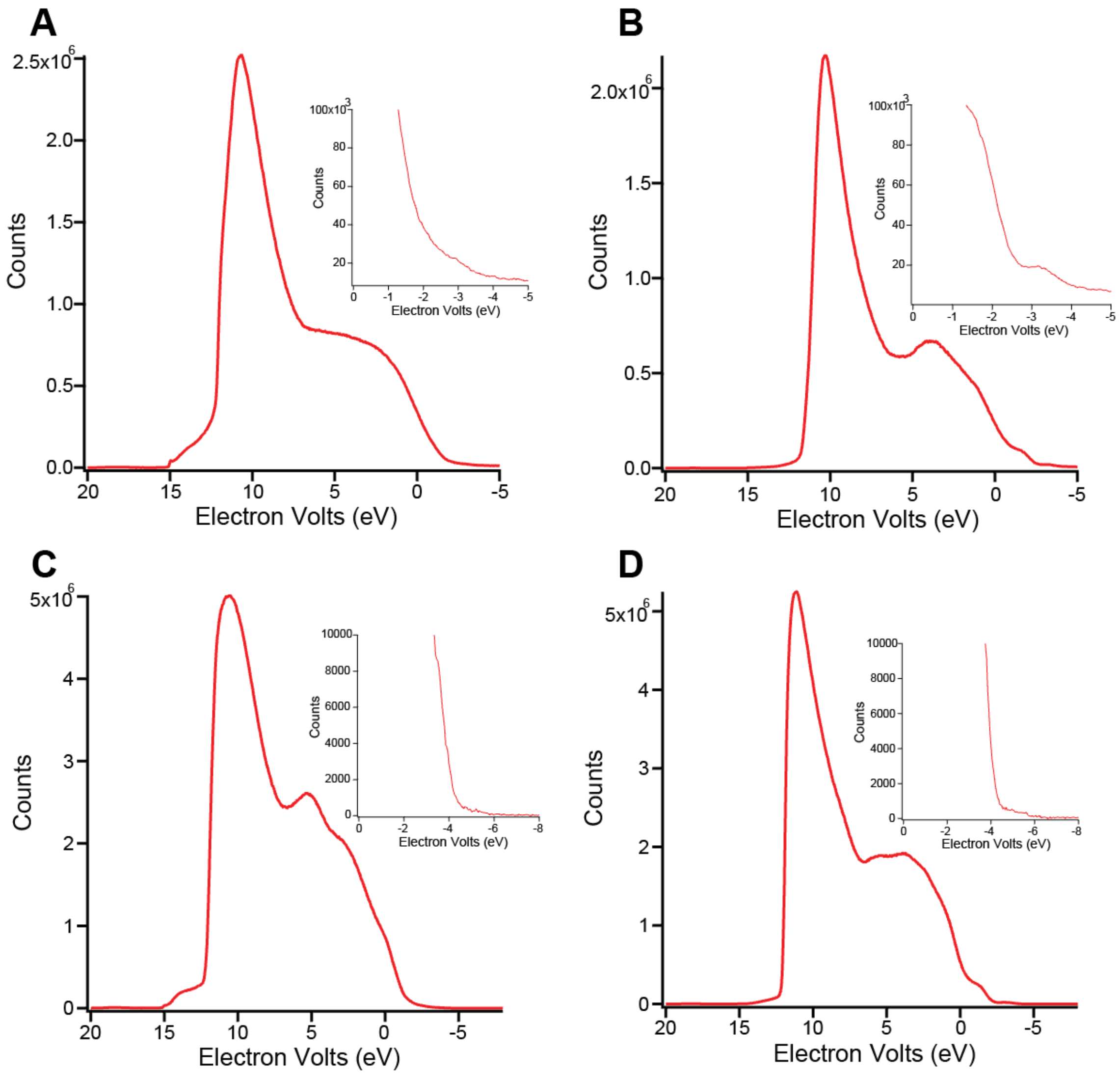

SI Figure 18: Ultraviolet photoelectron spectroscopy (UPS) of monomer and assembled nanostructures, with insets zooming in on low energy region used for HOMO level determination. A) UPS spectrum of **1**. B) UPS spectrum of **2**. C) UPS spectrum of **1'**. D) UPS spectrum of **2'**.

Cryogenic transmission electron microscopy (Cryo-TEM)

Cryo-TEM imaging was performed on a JEOL 1230 microscope, operating at 100 kV. A 6.5 μL droplet was placed on either a lacey carbon copper grid or C-flat grid (CF-4/2-4C-25, Electron Microscopy Science). The grid was held by tweezers mounted on a Vitrobot Mark IV equipped with a controlled humidity and temperature environment. The temperature was set to 24°C and humidity was held at 90%. The specimen was blotted and plunged into a liquid ethane reservoir cooled by liquid nitrogen. The vitrified samples were transferred to a Gatan 626 cryo-holder through a cryo-transfer stage cooled by liquid nitrogen. During observation of the vitrified samples, the cryo-holder temperature was maintained below -180°C. The images were recorded with a CCD camera.

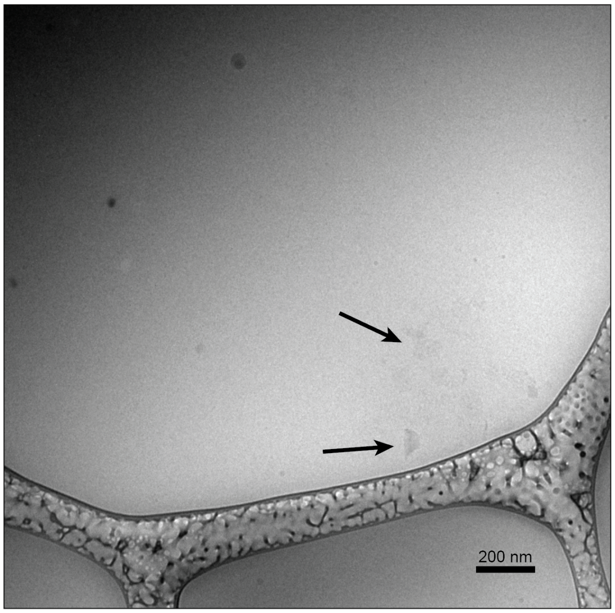

SI Figure 19: Cryo-TEM image of **1'** at 7.25 mM in aqueous solution.

Molecular Dynamics Simulations:

Classical full atom molecular dynamics simulations were performed to investigate the self-assembled nanostructures from hydroxyl PMI at different protonation state. The interaction potential was adapted from standard OPLS-AA/L force field.[5-7] The simulations were carried out with GROMACS 4.5.5.[8] The assembly kinetics was beyond the capability of classical MD[9, 10], the equilibrium assembly structures were therefore achieved through relaxation of pre-arranged crystalline phase, rather than from complete monomeric self-assembly.

Explicit solvent and ion models were used. Water molecules were represented with SPC/E model for good bulk dynamics and relatively cheap computational cost.[8] Ions were modeled by parametrizations in OPLS-AA/L force field.[7]

Five different systems were set up based on experimental configurations: (1) protonated hydroxyl group without extra salt (2) protonated hydroxyl group with NaCl extra salt at 7:1 molar ratio (3) deprotonated hydroxyl group without extra salt (4) deprotonated hydroxyl group with NaCl extra salt at 7:1 molar ratio (5) deprotonated hydroxyl group with $CaCl_2$ extra salt at 6:1 molar ratio. In each system, 60 PMI molecules were arranged into a one crystalline flat ribbon (Fig 1 a), where a unit cell of 0.36 nm x 9 nm was applied (Fig 1 b). The dimension of the ribbon 3.6 nm x 3.6 nm, or 6 x 10 molecules. The ribbon was solvated with explicit water molecules in a simulation box of 8 nm x 8 nm x 8 nm; 60 or 120 Na^+ ions were always added as counterions, depending on the protonation state of hydroxyl group. Additional NaCl or $CaCl_2$ ion pairs were added to achieve the correct salt concentration.

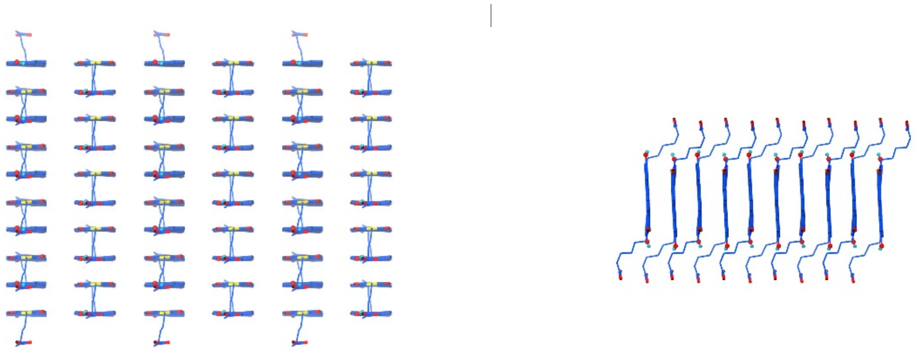

Initial Configuration

Energy minimization was performed with steep algorithm for 2500 steps or best convergence to resolve steric collapse. A total of 5 equilibration simulations were carried out to relax solvent and ions subsequently. The PMI molecules were position restrained throughout all equilibration phases to retain crystallinity. The force constant for this harmonic restrain was 1000 kJ/nm^2. Constant temperature and volume ensemble (NVT) was first used to contain fluctuation; constant temperature and pressure ensemble (NPT) was then adapted to achieve optimal solvent density. The simulation times for each equilibration phase were 10 ps NVT, 10 ps NPT, 100 ps NPT, 500 ps NPT and 500 ps NPT respectively. Integration time steps and sophistication of thermodynamic coupling algorithm were gradually increased.

After equilibration, 10 ns simulation under NPT ensemble was performed for each system for structural and dynamics analysis. The simulation was kept at 298 K through Nose-Hoover thermostat and 1 bar via anisotropic Parrinello-Rahman barostat. The characteristic time for the thermostat was 0.5 ps and 4.0 ps for the barostat. The compressibility was 4.5x10^{-5} bar^{-1}. Nonbonding interactions were calculated with a neighbor searching list at cutoff 1.2 nm. The trajectory was updated every 20 fs. Full electrostatics interactions were computed with Particle Mesh Ewald (PME) method, with a space cutoff at 1.2 nm and a Fourier grid width 0.12 nm. No atomic coordinates were constrained. Hydrogen bonds were held rigid with LINCS algorithm to allow 2 fs time steps. The stability of simulation as verified by potential and pressure calculations.

Calculation of direction vectors

Two direction vectors were defined for each PMI molecule. The normal vector was defined as the vector perpendicular to the PMI chromophoric plane; the in-plane vector was defined in the same plane of PMI chromophoric plane, pointing from the center of the plane to the nitrogen atom of the imide (Figure 5B). These two vectors were perpendicular to each other and accounted for different types of rotation of each PMI molecule.

To account for the level of crystallinity of overall PMI self-assembly, the angle deviations of individual direction vectors from their average/assembly value were calculated for each PMI. The angle deviation was calculated according to the matlab function $angle = atan2(norm(cross(a, b), dot(a, b)))$, where the angle was restricted

to a range from 0 to pi radians. The calculated angle deviations were plotted into histograms, where the peak location and spread of the histogram accounted the level of orderliness of PMI self-assembly.

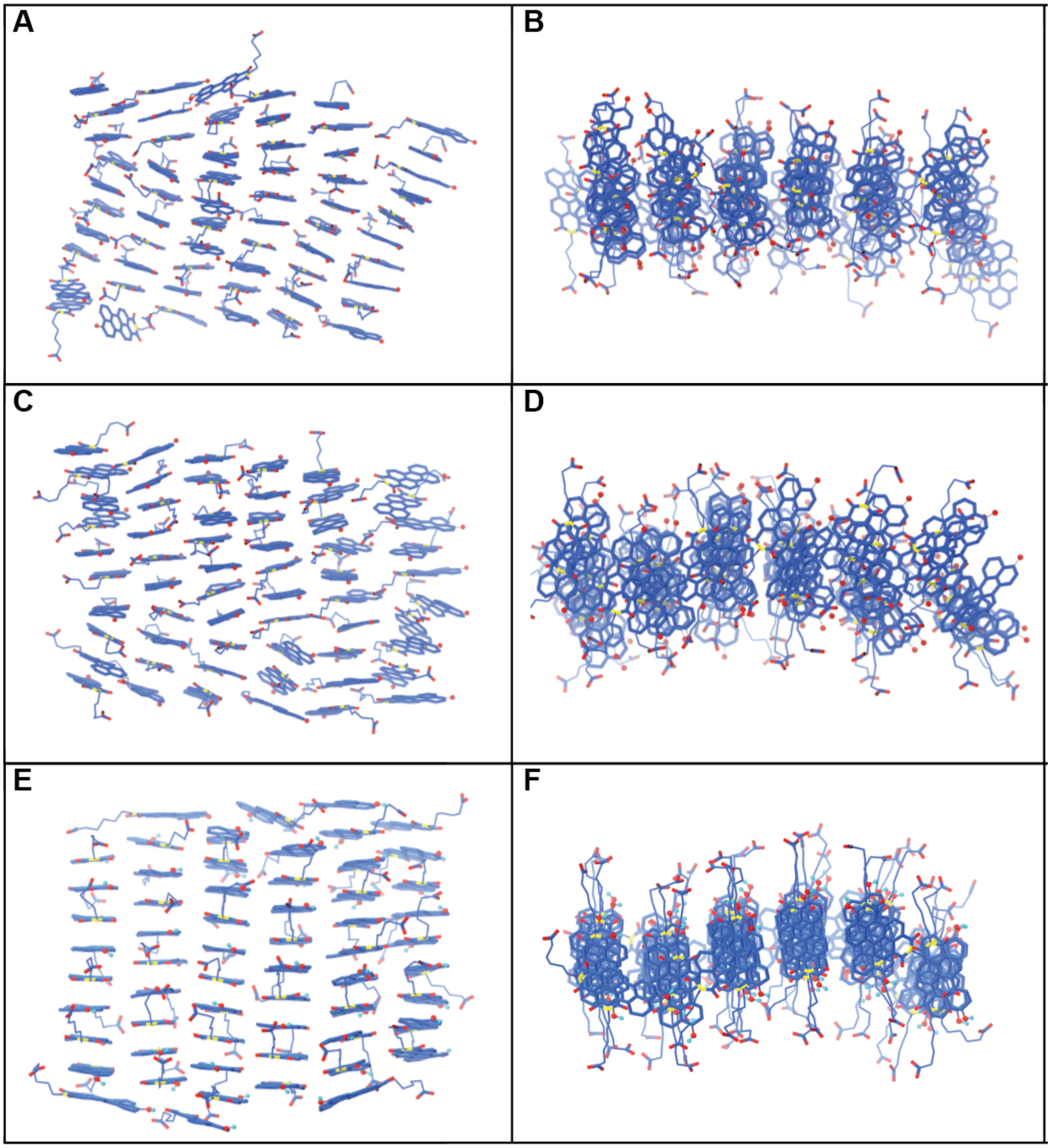

SI Figure 20: Snapshots of MD simulations on pre-assembled PMI ribbons. 60 total PMI molecules arranged in an anti-parallel packing order with a 0.36 x 0.9 nm unit-cell as a 6 x 10 mesh inside of a 8 x 8 x 8 simulation box. Simulations were conducted using a NPT ensemble without any constrains. A) Top view of **1'** with 6 eq of $CaCl_2$. B) Side view of **1'** with 6 eq of $CaCl_2$. C) Top view of **1'** with 7 eq of NaCl. D) Side view of **1'** with 7 eq of NaCl. E) Tope view of **1** with 7 eq NaCl. F) Side view of **1** with 7 eq NaCl.

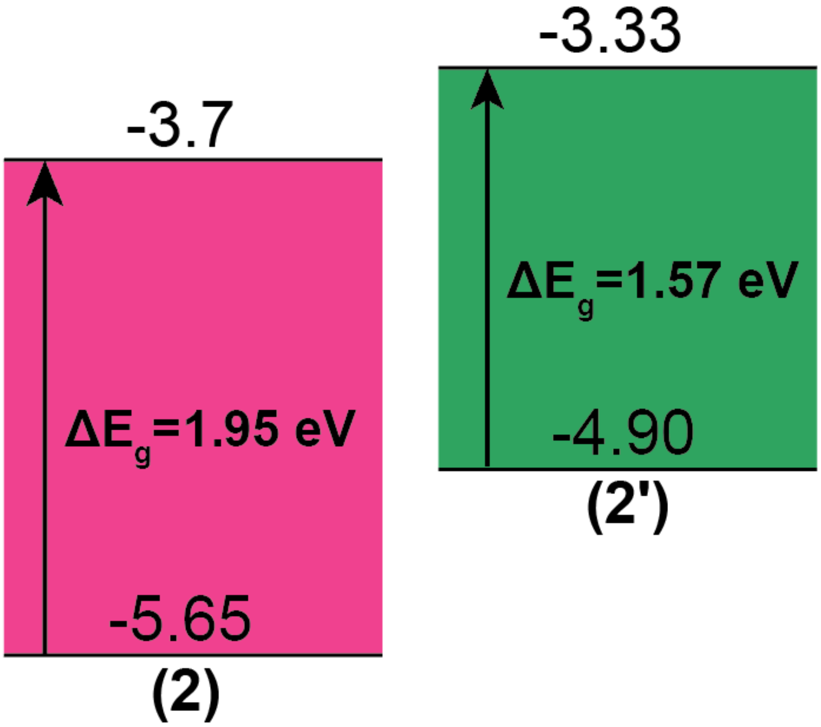

SI Figure 21: HOMO/ LUMO energy level diagram for compound **2** and **2'** determined using UPS and UV-Vis.

DFT Calculations:

DFT calculations were performed on the Firefly quantum calculation package which is partially based on the GAMESS (US) source code[11]. We used the B3LYP/6-311++G(d,p) hybrid function and basis set, respectively, with the polarization functions applied to oxygen and metal atoms. The input file construction and output file visualization was performed using the Facio software.[12] Simulations of the deprotonated PMI-O^- were more difficult because DFT is not well suited to handling anionic species, so sodium counterions were included in the simulation. The alkyl tail and carboxylic acid groups were omitted from the calculations because the node present at the imide nitrogen prevents any group attached there from changing the electronic structure and in order to decrease run time.

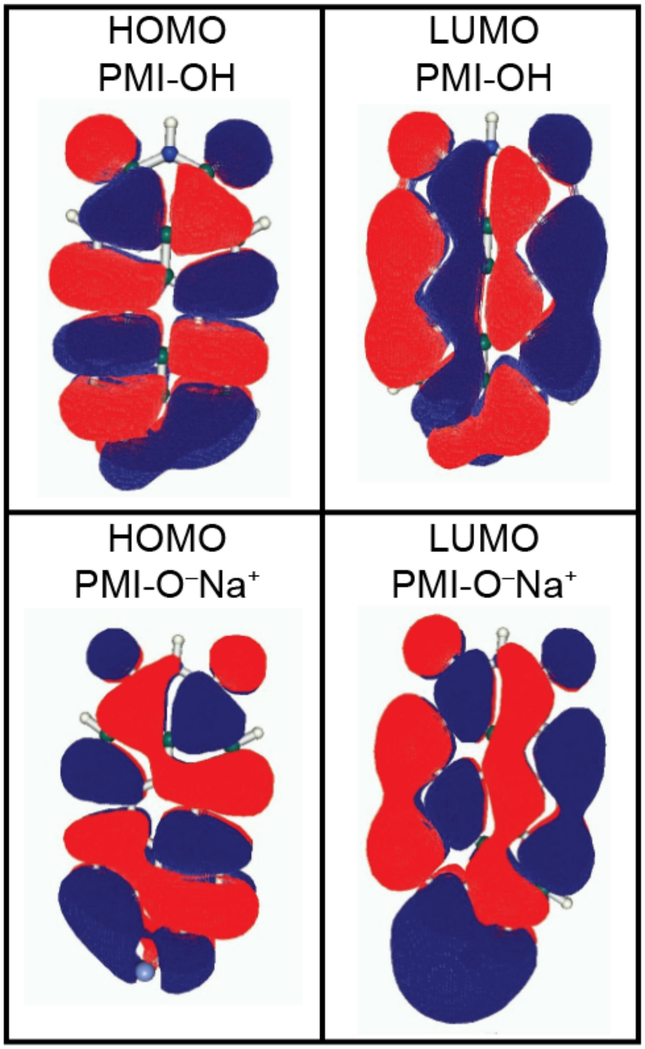

SI Figure 22: Schematic representation of the HOMO and LUMO orbitals of the protonated PMI and deprotonated PMI (with sodium counter ion) core. Imide substituent has been replaced with hydrogen atom for simulations. B3LYP/6-311++G(d,p)

^{1}H and ^{13}C NMR Spectra:

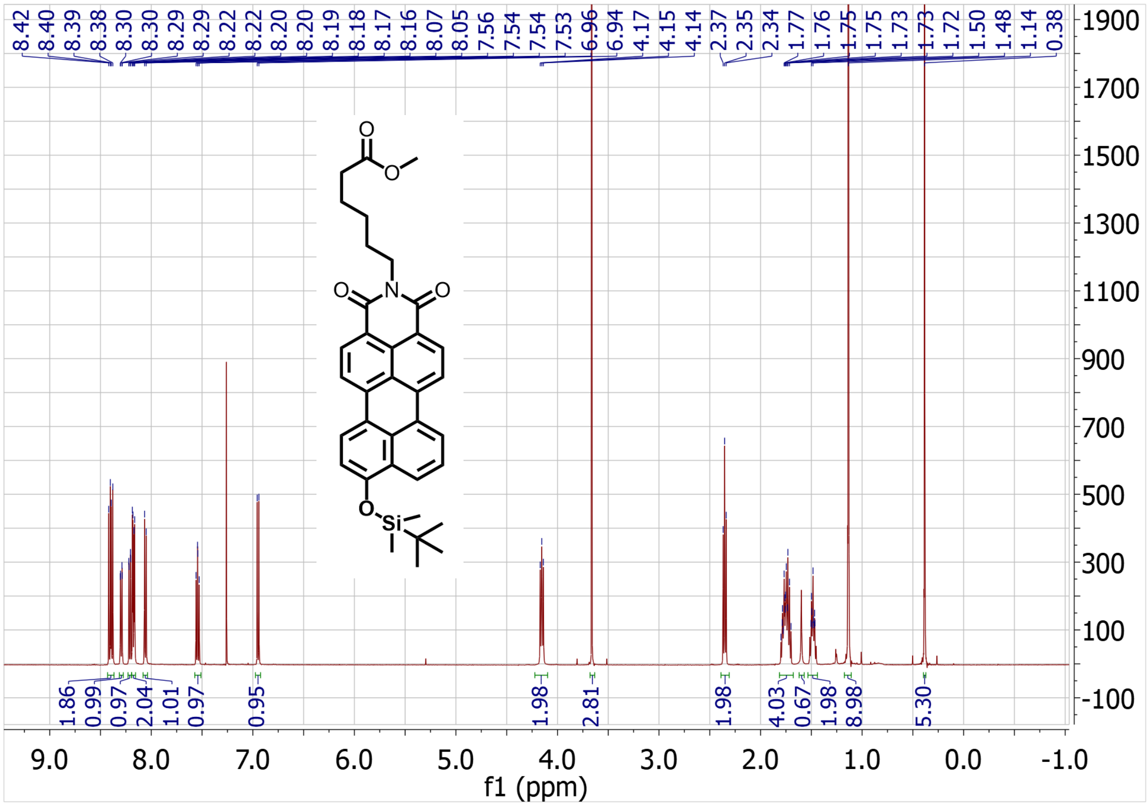

SI Figure 23: ^{1}H NMR spectrum of compound **S2** (500 MHz, $CDCl_3$, 25 °C)

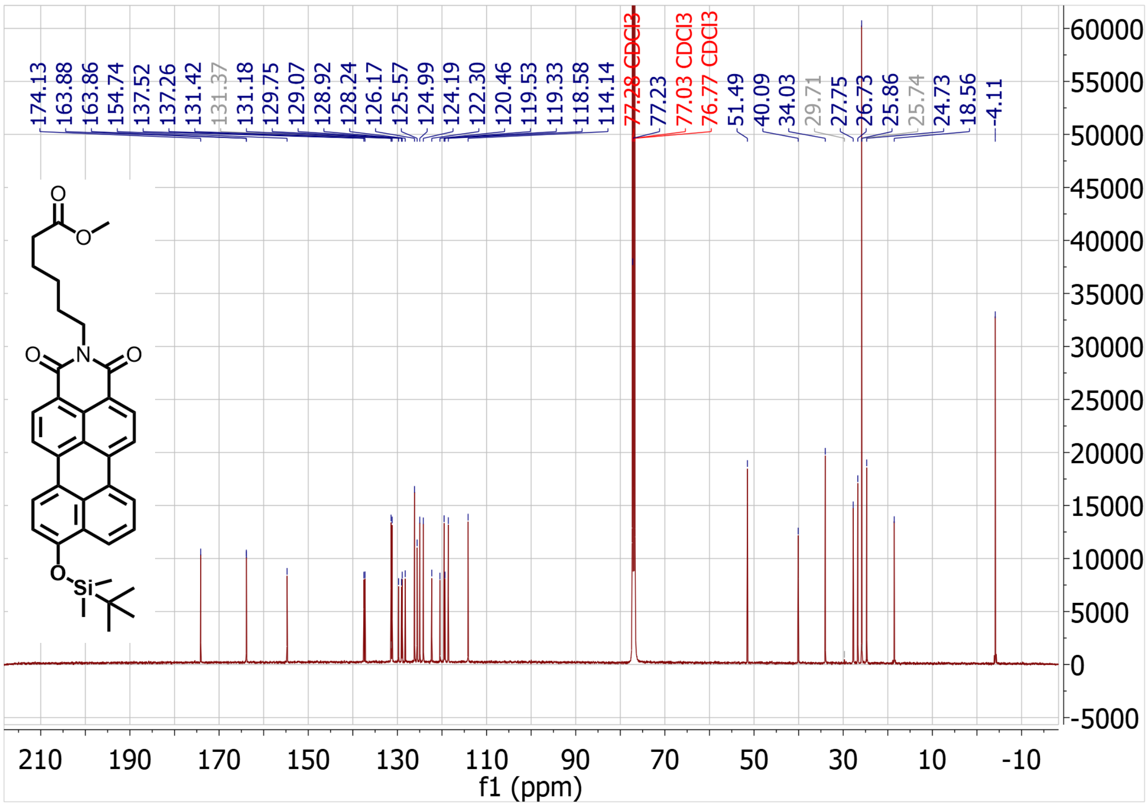

SI Figure 24: ^{13}C NMR spectrum of compound **S2** (125 MHz, $CDCl_3$, 25 °C)

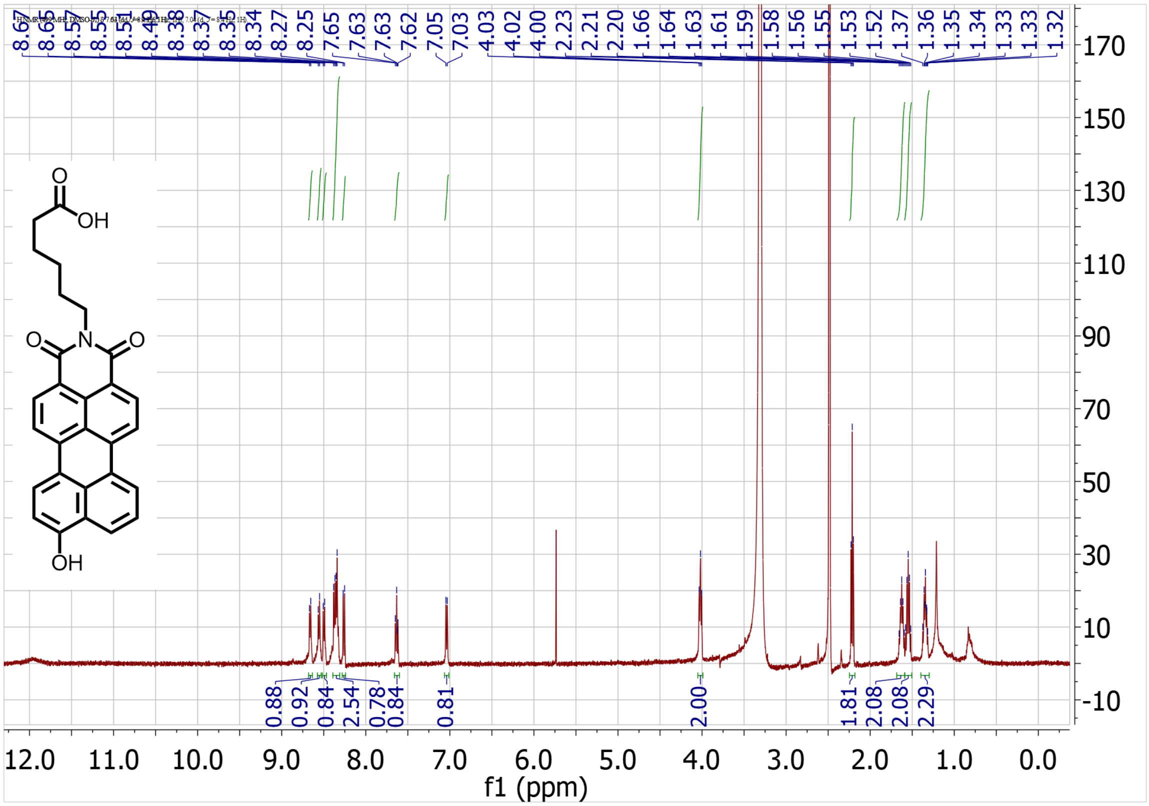

SI Figure 25: ^{1}H NMR spectrum of compound **1** (500 MHz, d_6-DMSO, 25 °C)

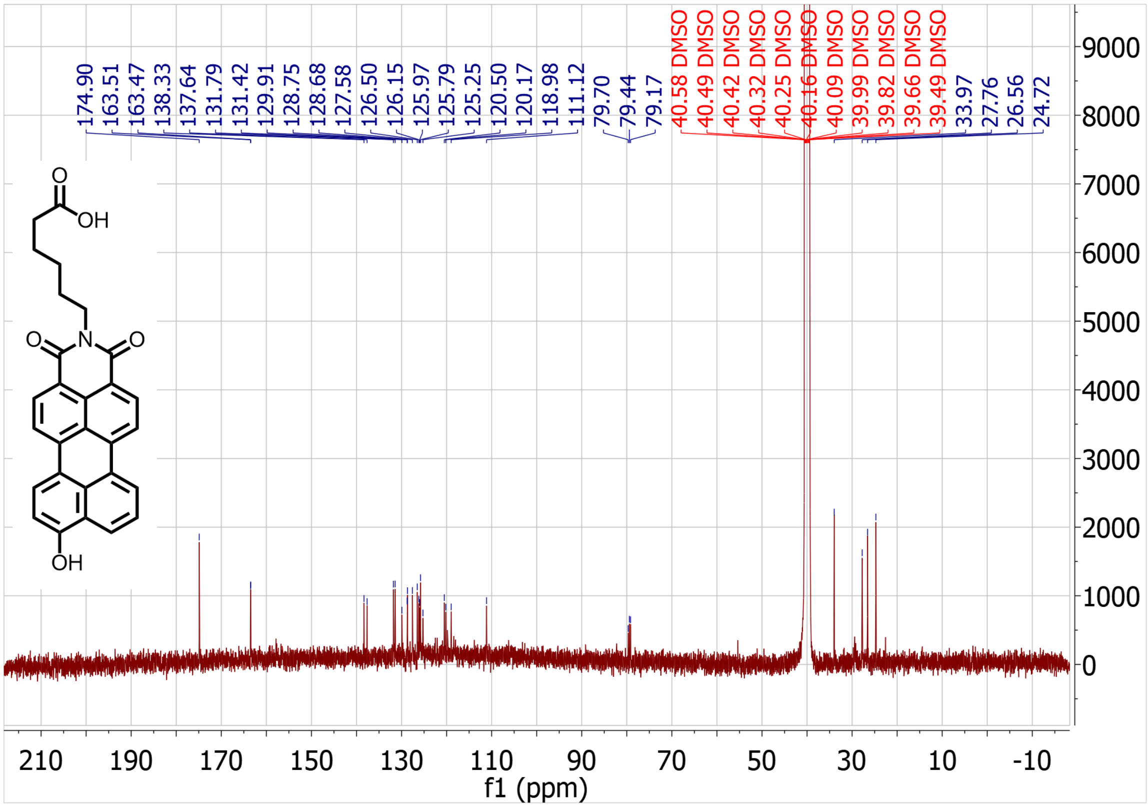

SI Figure 26: ^{13}C NMR spectrum of compound **1** (125 MHz, d_6-DMSO, 25 °C)

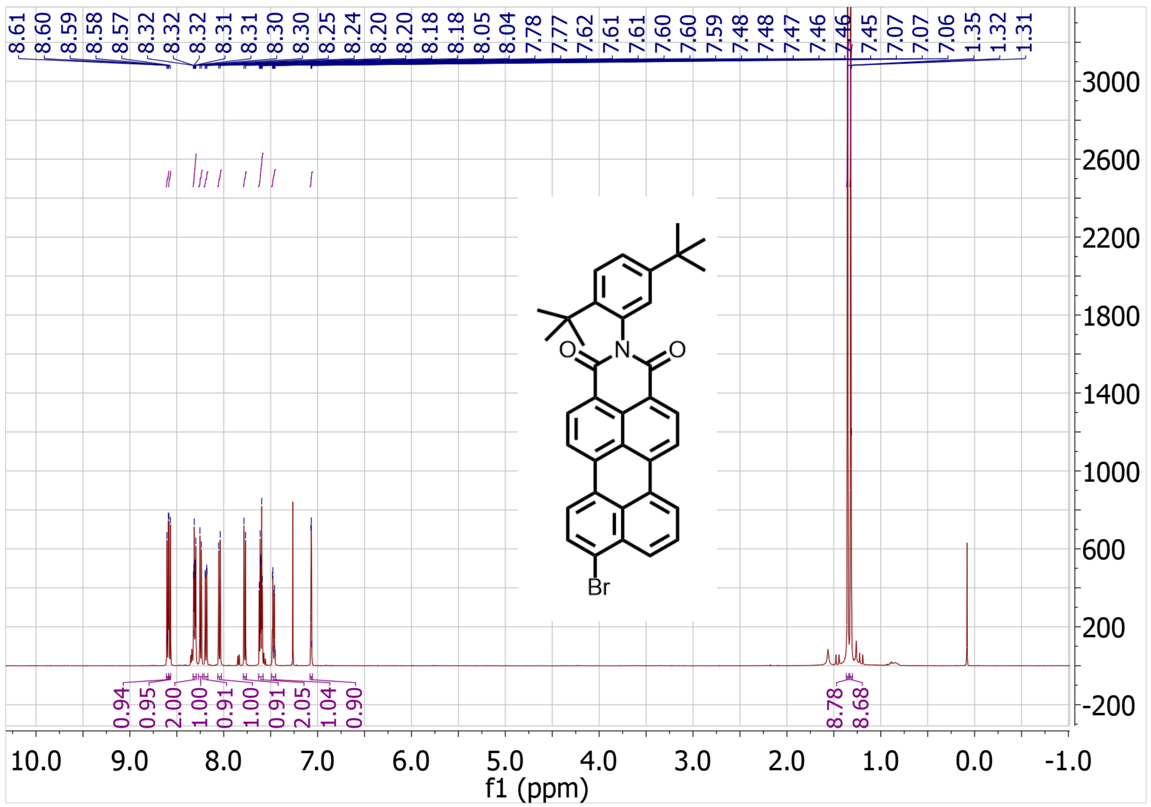

SI Figure 27: ^{1}H NMR spectrum of compound **S4** (500 MHz, CDCL$_3$, 25 °C)

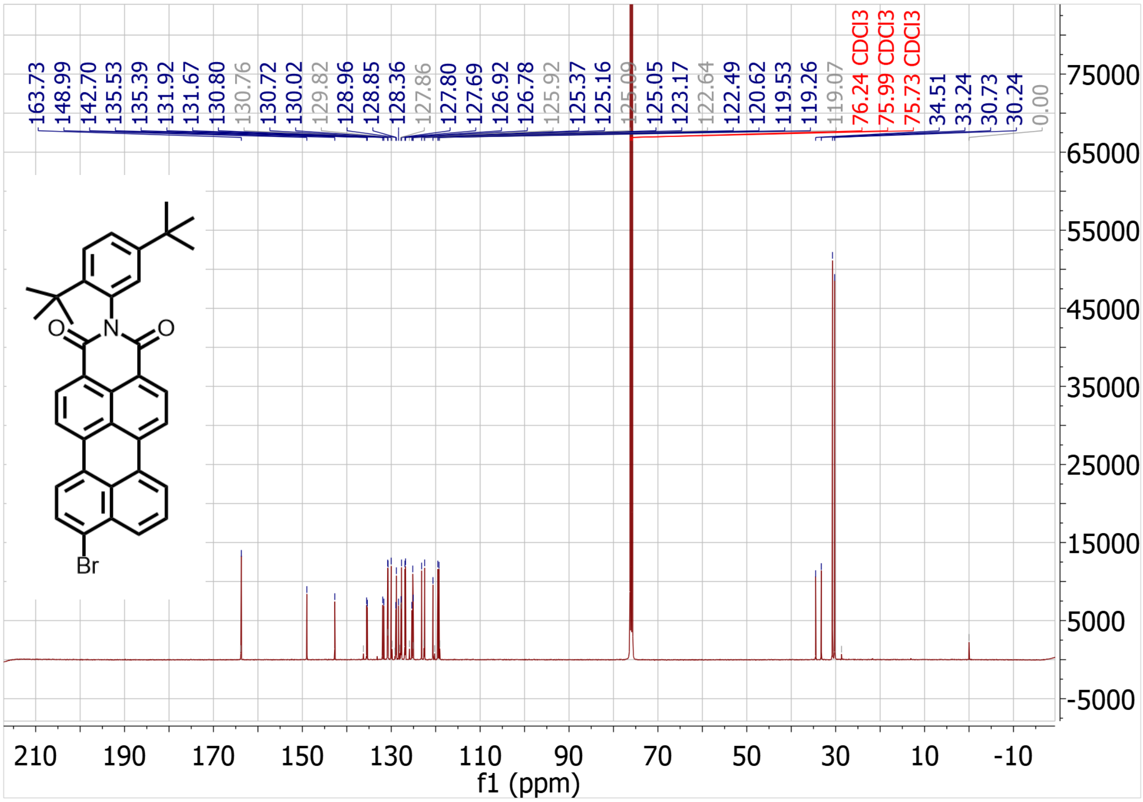

SI Figure 28: ^{13}C NMR spectrum of compound **S4** (125 MHz, $CDCl_3$, 25 °C)

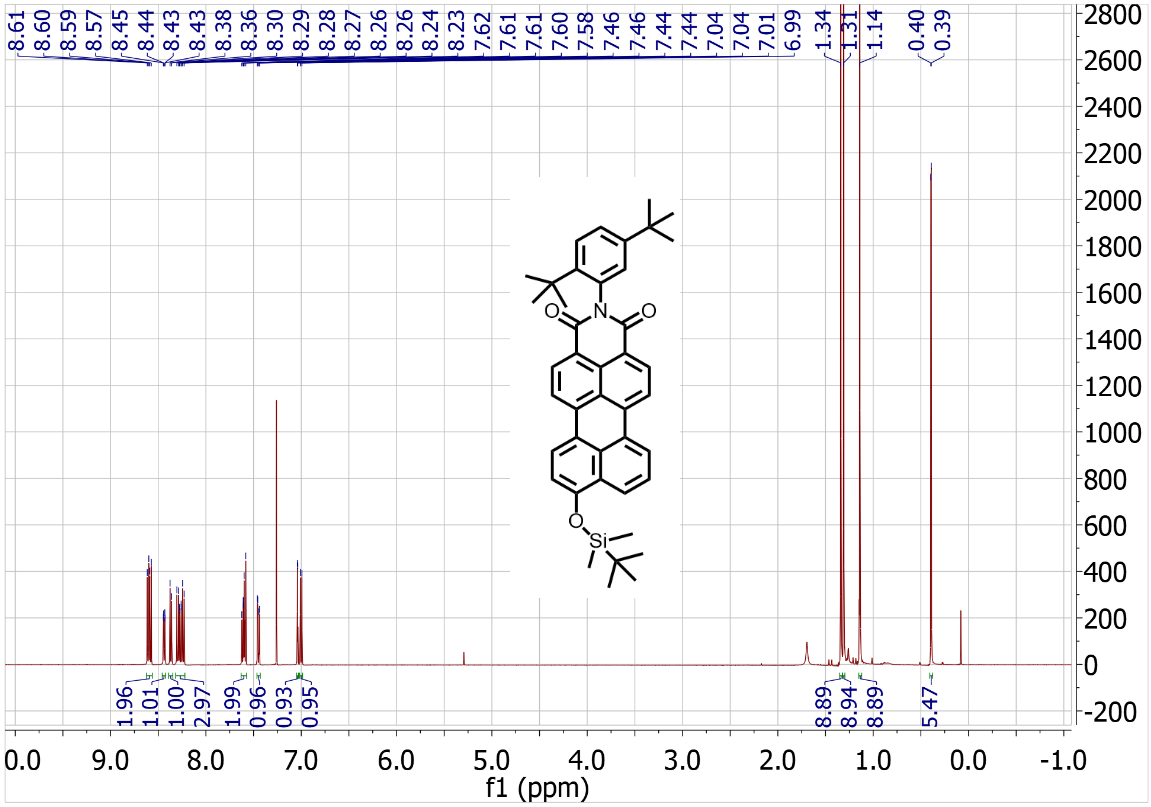

SI Figure 29: ^{1}H NMR spectrum of compound **S5** (500 MHz, $CDCL_3$, 25 °C)

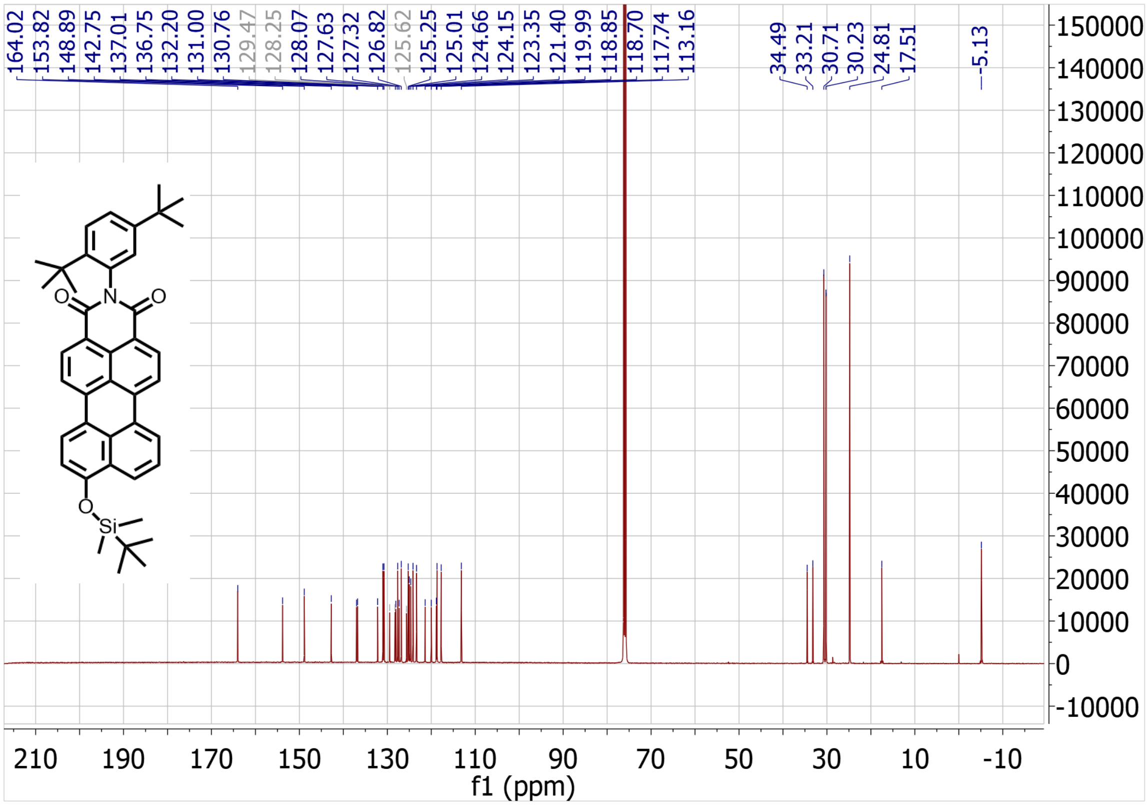

SI Figure 30: ^{13}C NMR spectrum of compound **S5** (125 MHz, $CDCL_3$, 25 °C)

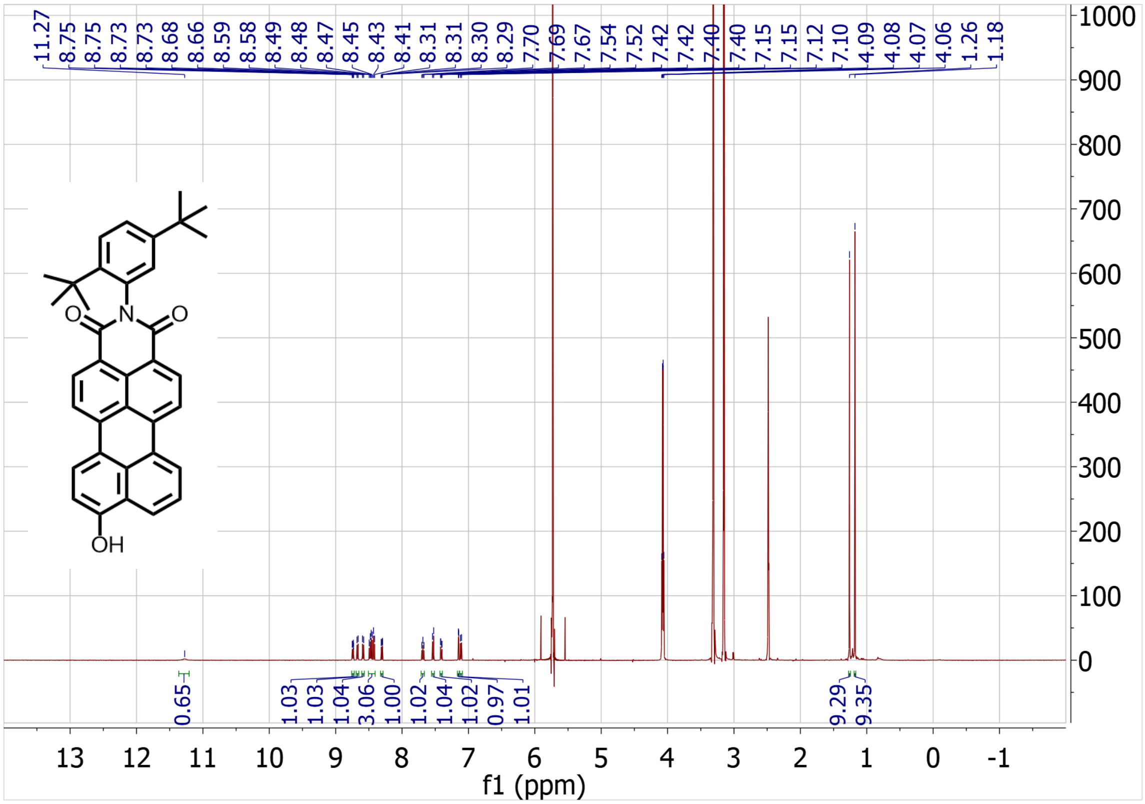

SI Figure 31: ^{1}H NMR spectrum of compound **2** (500 MHz, d_6-DMSO, 25 °C)

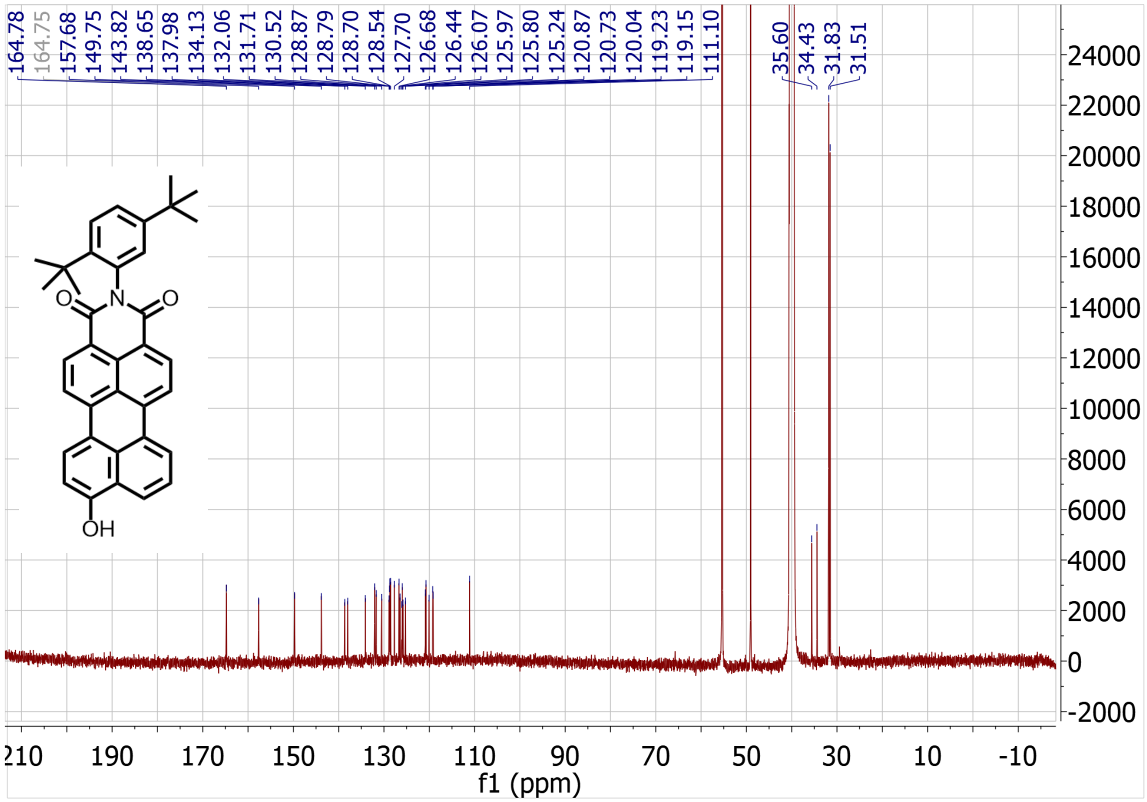

SI Figure 32: ^{13}C NMR spectrum of compound **2** (125 MHz, d_6-DMSO, 25 °C)

References:

1. R. V. Kazantsev, A. J. Dannenhoffer, A. S. Weingarten, B. T. Phelan, B. Harutyunyan, T. Aytun, A. Narayanan, D. J. Fairfield, J. Boekhoven, H. Sai, A. Senesi, P. I. O'Dogherty, L. C. Palmer, M. J. Bedzyk, M. R. Wasielewski and S. I. Stupp, *J. Am. Chem. Soc.*, 2017, **139**, 6120-6127.
2. J. Kibsgaard, T. F. Jaramillo and F. Besenbacher, *Nat Chem*, 2014, **6**, 248-253.
3. Z. Jiang, *J. Appl. Crystallogr.*, 2015, **48**, 917-926.
4. B. Harutyunyan, A. Dannenhoffer, S. Kewalramani, T. Aytun, D. J. Fairfield, S. I. Stupp and M. J. Bedzyk, *J. Phys. Chem. C*, 2017, **121**, 1047-1054.
5. C. Caleman, P. J. van Maaren, M. Hong, J. S. Hub, L. T. Costa and D. van der Spoel, *Journal of chemical theory and computation*, 2011, **8**, 61-74.
6. C. L. Yaws, *Thermophysical properties of chemicals and hydrocarbons*, William Andrew, 2008.
7. W. L. Jorgensen, D. S. Maxwell and J. Tirado-Rives, *J. Am. Chem. Soc*, 1996, **118**, 11225-11236.
8. S. Pronk, S. Páll, R. Schulz, P. Larsson, P. Bjelkmar, R. Apostolov, M. R. Shirts, J. C. Smith, P. M. Kasson and D. van der Spoel, *Bioinformatics*, 2013, **29**, 845-854.
9. P. Mark and L. Nilsson, *The Journal of Physical Chemistry A*, 2001, **105**, 9954-9960.
10. I. Autiero, M. Saviano and E. Langella, *PCCP*, 2014, **16**, 1868-1874.
11. K. Ishimura, L. McMurchie, E. Davidson and J. Rys, *J. Comput. Chem*, 1993, **14**, 1347-1363.
12. M. Suenaga, *J. Comput. Chem. Jpn*, 2005, **4**, 25-32.
